# Disinhibition of olfactory bulb granule cells accelerates odour discrimination in mice

**DOI:** 10.1038/ncomms9950

**Published:** 2015-11-23

**Authors:** Daniel Nunes, Thomas Kuner

**Affiliations:** 1 Department of Functional Neuroanatomy, Institute for Anatomy and Cell Biology, Heidelberg University, Im Neuenheimer Feld 307, 69120 Heidelberg, Germany

## Abstract

Granule cells are the dominant cell type of the olfactory bulb inhibiting mitral and tufted cells via dendrodendritic synapses; yet the factors regulating the strength of their inhibitory output, and, therefore, their impact on odour discrimination, remain unknown. Here we show that GABA_A_R β3-subunits are distributed in a somatodendritic pattern, mostly sparing the large granule cell spines also known as gemmules. Granule cell-selective deletion of β3-subunits nearly abolishes spontaneous and muscimol-induced currents mediated by GABA_A_ receptors in granule cells, yet recurrent inhibition of mitral cells is strongly enhanced. Mice with disinhibited granule cells require less time to discriminate both dissimilar as well as highly similar odourants, while discrimination learning remains unaffected. Hence, granule cells are controlled by an inhibitory drive that in turn tunes mitral cell inhibition. As a consequence, the olfactory bulb inhibitory network adjusts the speed of early sensory processing.

## Abstract (web summary)

How odour discrimination is influenced by granule cells in the olfactory bulb is poorly understood. Here, the authors show that disinhibition of granule cells in mice increases mitral cell inhibition and accelerates odour discrimination time, independent of odour similarity.

## Graphical abstract

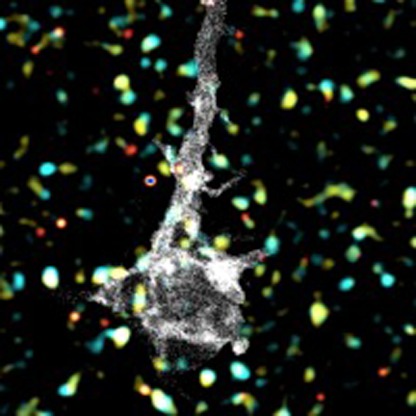

Granule cells (GCs) are key players for the early processing of odour information in the olfactory bulb (OB). These interneurons are exclusively GABAergic^1^ and account for the second layer of inhibition within the OB, dominating the OB circuitry^2^,^3^,^4^,^5^. GCs are involved in synchronization and establishment of slow temporal patterns of mitral cells (MCs)^6^,^7^,^8^, contrast enhancement for spatial representations of odours^8^,^9^,^10^ and sharpening of activity onset in the OB network^11^,^12^. These are features of recurrent and lateral inhibition in the OB, mediated by the reciprocal synapse established between the GCs and MCs^13^,^14^,^15^. This synapse has been characterized at molecular, physiological and behavioural levels^11^,^16^,^17^,^18^,^19^.

While the function of GCs with regard to MC inhibition is well studied on different levels, it remained less clear how GC activity itself is regulated and how it affects odour discrimination behaviour. Previous work described that GC activity is refined by inhibition mediated by monosynaptic inputs from Blanes cells, large stellate-shaped GABAergic interneurons present in the GC layer (GCL) that fire persistently on glomerular stimulation^20^,^21^,^22^,^23^. *In situ* hybridization studies have shown that GCs express all subunits necessary to generate both tonic and phasic inhibition^24^,^25^, although GCs express only the β3-subunit of the β-subunit subfamily^7^,^25^,^26^. GABA_A_ receptors (GABA_A_Rs) containing the β3-subunit are major players in the coordination of network oscillations and are involved in synaptic inhibition^27^,^28^. Genetic deletion of the β3-subunit impairs odour discrimination behaviour and learning in mammals^7^. However, using mice globally lacking β3-subunits it is difficult to attribute the observed behavioural phenotype to a specific mechanism, cell type or brain region. Any cell-type-specific or even OB-specific conclusions from global β3-knockouts are limited because mitral and tufted cells in the OB express the β3-subunit as well^25^,^26^ and β3 is expressed in other brain regions potentially involved in odour learning, discrimination and decision-making.

How does inhibition of GCs affect MC inhibition in the main OB? Where does this inhibition take place in the GC? How does GC inhibition affect odour discrimination speed and accuracy? We addressed these questions using a conditional mouse line with loxP sites flanking the exon 3 of the *GABRB3* gene^29^ to selectively abolish GABA_A_R-dependent inhibition in GCs by viral expression of Cre-recombinase in the GCL^19^. Our immunohistochemistry data show a clustered distribution of β3-subunit-containing GABA_A_Rs in the somata and dendritic stems of GCs representing GABAergic synapses. At the physiological level, the phasic inhibition mediated by these synapses strengthens MC inhibition. Finally, we use the go/no-go operant-conditioning paradigm to show that specific deletion of the β3-subunit in the GCL accelerates odour discrimination times (ODTs) for monomolecular odours and highly similar mixtures, but leaves discrimination learning unaffected.

## Results

### Clustered distribution of β3-containing GABA_A_Rs in GCs

**Figure 1:**
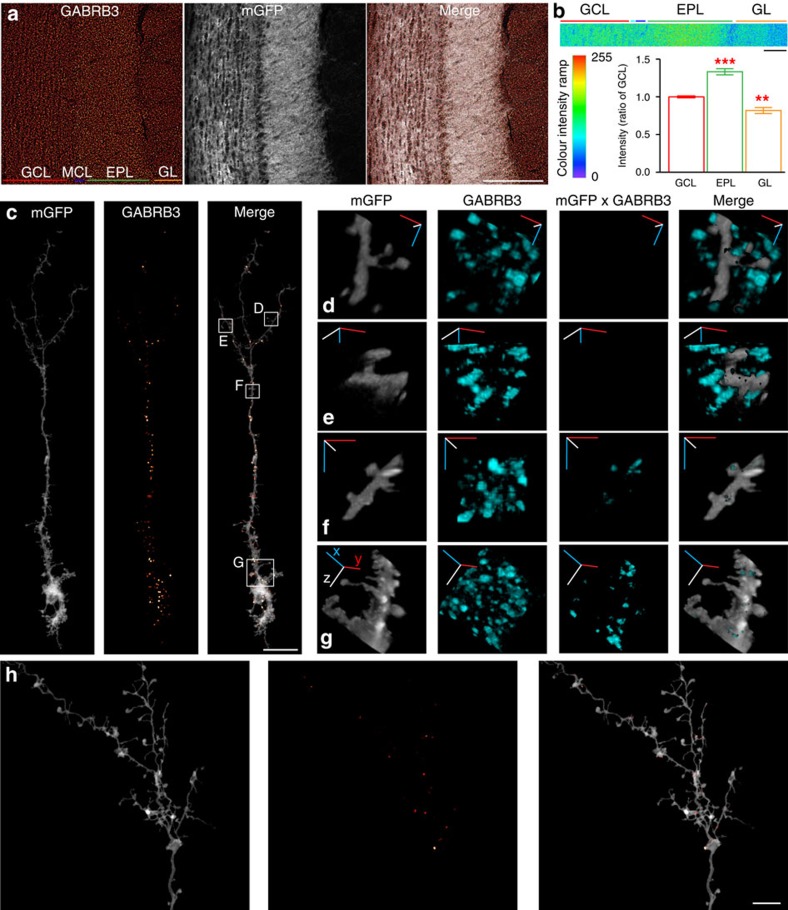
Clustered distribution of the β3-subunit. (**a**) Immunostaining using a β3-subunit-selective antibody (red hot colour palette) in horizontal OB slices infected with rAAV-mGFP (grey). The images represent single confocal frames. From left to right, GCL, MC layer (MCL), EPL and glomerular layer (GL). Scale bar, 200 μm. (**b**) Abundance of β3-subunit immunosignal in the EPL and GL relative to the GCL. Representative image used to quantify the grey-scale intensity values (here using the rainbow colour palette). See Methods for further details. Ratios relative to GCL in EPL (1.33±0.04; *n*=16; ***P<0.001), GL (0.82±0.04; *n*=16; **P<0.01; Bonferroni's multiple comparison test). The mean ratios±s.e.m. Scale bar, 58 μm. (**c**) Distribution of β3-subunit clusters (red hot colour palette) within a single GC (grey). Images represent maximum-intensity projections of a confocal stack extending over 70 image frames, after frame-by-frame multiplication as illustrated in Supplementary Fig. 1C–F. Grey-scale levels adjusted for display. Scale bar, 0.25 μm. (**d**–**g**) 3D reconstructions of regions shown in **c**. 3D reconstruction procedure is illustrated in Supplementary Figs 2 and 3. The cartesian axes *x* (blue), *y* (red) and *z* (white) show the rotation of the reconstructed region from origin. The width of the image corresponds to ∼12.5 μm in **d**–**f** and 25 μm in **g**. Panels: mGF*P*, membrane-labelled GC; GABRB3, all immunosignals in the respective volume; mGFPxGABRB3, immunosignals residing inside the GC; merge, **d**,**e** show all GABRB3 immunosignals surrounding the GC; **f**,**g** show GABRB3 immunosignals residing within the GC. (**h**) Distribution of β3-subunit clusters (red hot colour palette) within the distal dendritic tree (grey). The images represent maximum-intensity projections of a confocal stack extending over 124 image frames. Grey-scale levels adjusted for display. Scale bar, 10 μm.

We first examined the expression pattern of the β3-subunit in horizontal sections of the OB using a β3-selective antibody. Consistent with previous results^7^,^30^, immunolabelling was found in all layers of the OB (Fig. 1a), but most prominently in the external plexiform layer (EPL, Fig. 1b). Viral labelling of GCs with membrane-bound green fluorescent protein (mGFP) reveals β3-subunit immunolabelling throughout the spatial domain occupied by the GCs (Fig. 1a). To visualize the expression and distribution of the β3-subunit within GCs, single GCs were labelled by sparsely expressing mGFP in the GCL (Supplementary Fig. 1A,B). To achieve this, rAAV-DIO-mGFP together with 1:1,000 diluted rAAV-Cre particles were stereotaxically delivered to the GCL of wild-type mice. After 3 weeks of expression, the OB tissue was stained with antibodies directed against the β3-subunit. Using the three-dimensonal (3D)-immunohistochemistry approach^31^, a strategy to selectively visualize proteins expressed within a structure of interest (illustrated in Supplementary Fig. 1C–F), we found a clustered distribution of the β3-subunit in GCs (Fig. 1c–h). These clusters show a preferred localization at the soma and dendritic shaft (Fig. 1c), with only low abundance in the distal dendritic tree of GCs (Fig. 1c,h). Magnified 3D reconstructions of the regions indicated in Fig. 1c further illustrate this distribution pattern (Fig. 1d–g). The two apical regions shown in panels d and e reveal dense β3-immunoreactive clusters; yet, none of these are positioned within the apical GC dendrite. Consistent with their localization in the EPL, these clusters represent inhibitory synaptic contacts of the MC lateral dendrites, known to abundantly express β3-subunits. In contrast, the two regions selected from a distal part of the primary dendrite (F) and the proximal dendrite at the junction to the soma (G) contain multiple β3-immunoreactive clusters (Fig. 1f,g). β3-positive immunoreactivity was mostly absent from GC gemmules (Fig. 1c–h). Identical results were obtained in a total of five cells taken from three mice analysed with this approach. In summary, these results predict that inhibitory inputs at the soma and dendritic shaft produce a rather global inhibition of GCs, while a local, gemmule-specific inhibition appears unlikely. Furthermore, the clustered distribution is consistent with a synaptic localization of the β3-subunit-containing GABA_A_Rs in GCs.

**Figure 2:**
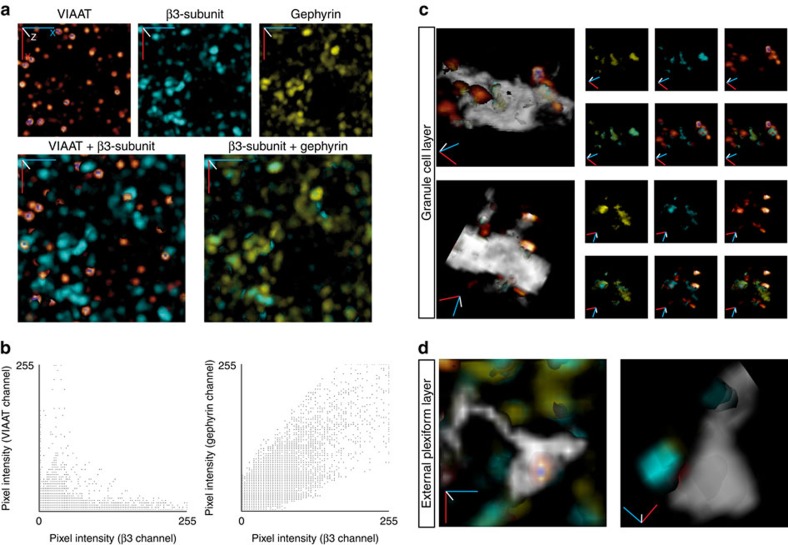
Synaptic localization of the β3-subunit, Gephyrin and GABA vesicles. (**a**) 3D projections of the immunohistochemical detection of VIAAT (glow over palette), β3-subunit (cyan) and gephyrin (yellow) showing its distribution in the GCL (upper panels). The β3-subunit clusters colocalize with the gephyrin clusters, while VIAAT clusters directly oppose β3-subunit clusters (lower panels). The Cartesian coordinates *x*(blue), *y* (red) and *z* (white) are at the origin, but do not represent scale bars because of non-isotropic pixel size. The projections are representative of a small area in the GCL (20 μm × 20 μm, pixel size=0.114 μm) extended over 50 image frames (total *z* depth of 7.5 μm). Three independent experiments were performed. (**b**) Scatter plots generated by ImageJ colocalization finder plugin from immunolocalization data shown in **a** (representative example of three independent experiments). The analysis presented here covers 378 β3-clusters, 454 gephyrin clusters and 188 VIAAT clusters. (**c**) 3D reconstructions showing the granule cell membrane (grey) of the soma (upper panel) and a portion of the dendrite (lower panel) with superpositon of the β3-subunit, gephyrin and VIAAT clusters on the left (colour code as in **a**). The upper row of small panels shows the signal residing within the GC for gephyrin and β3-subunit and the surrounding VIAAT signal. The lower row of small panels shows the colocalization of β3-subunit with gephyrin (left) or VIAAT (centre) or all (right). The 3D reconstruction procedure is illustrated in Supplementary Figs 2 and 3. Cartesian axes *x* (blue), *y* (red) and *z* (white), image width ∼10 μm. (**d**) 3D reconstructions of GC gemmules with superpositon of β3-subunit, gephyrin and VIAAT clusters (colour code as in **a**). Note that VIAAT is inside the GC (transparent grey), while β3-subunit and gephyrin colocalize outside but close to the gemule. Cartesian axes *x*(blue), *y* (red) and *z* (white), image width ∼10 μm.

To further support a synaptic localization of the β3-subunit-containing GABA_A_Rs, we co-labelled the postsynaptic scaffolding protein gephyrin and the presynaptic vesicular GABA transporter VIAAT (Fig. 2 and Supplementary Figs 2 and 3). Each of these stains exhibits a clustered distribution of the immunosignals, while the overlay of VIAAT and β3-signals reveals a lack of overlap yet close apposition of VIAAT clusters next to β3-clusters. In contrast, β3 and gephyrin signals widely overlap (Fig. 2a).

Pixel intensity-based colocalization analysis of the VIAAT and β3-immunosignals revealed no overlap (Fig. 2b, left; Pearson's correlation coefficient <0.2). The correlation found in the low-intensity range supports the notion that VIAAT and the β3-subunit are located in close proximity, with a few pixels of both signals overlapping because of the limited resolution of confocal microscopy. The same analysis of β3- and gephyrin immunosignals revealed a high degree of colocalization of the clusters (Fig. 2b, right; Pearson's correlation coefficient >0.8), suggesting that virtually all β3-containing GABA_A_Rs are inserted in the inhibitory postsynaptic matrix delineated by gephyrin.

These observations were further confirmed by detailed 3D analyses (Supplementary Fig. 3) of a small area of the cell body and dendritic shaft (Fig. 2c). The presynaptic vesicle cluster (visualized using the glow over palette) is located outside but in close proximity to the GC membrane (transparent grey). The postsynaptic β3 (cyan) and gephyrin (yellow) signals are located within the GC. This is further illustrated in the small panels showing the individual clusters, their pairwise and triple overlays. The reverse situation was found in the EPL. Here the β3 immunosignal was located outside of the labelled GC gemmule with an opposed cluster of inhibitory vesicles inside the GC gemmule (Fig. 2d). These results, found in three independent experiments, demonstrate that β3 immunosignals in the EPL predominantly reflect the postsynaptic component of dendrodendritic synapses in MCs, while in the GCL β3 immunosignals report postsynaptic sites in GCs.

Taken together, these data demonstrate the synaptic localization of β3-subunit-containing GABA_A_Rs in GCs, predicting that inhibition of GCs will be exclusively synaptic.

### GC layer-specific deletion of the β3-subunit

**Figure 3:**
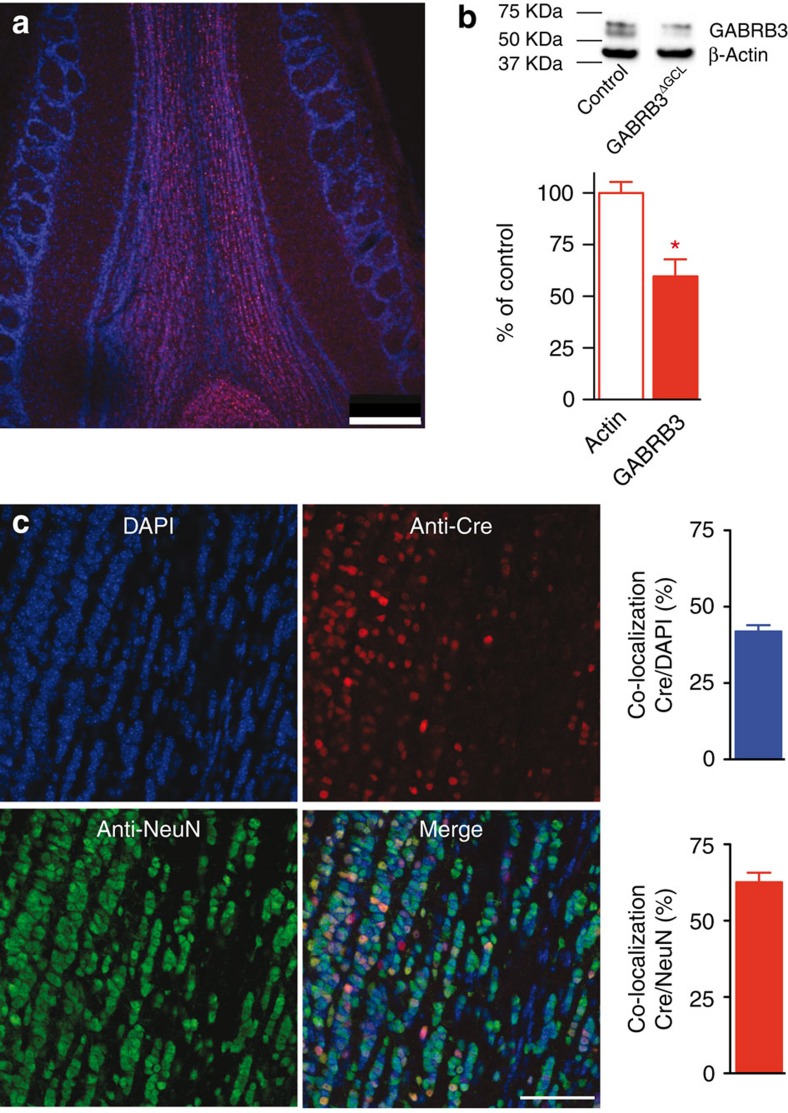
Quantification of β3-subunit deletion in the GCL. (**a**) Single-frame confocal overview of a horizontal section showing anti-Cre (magenta) and DAPI (blue) staining in a representative example of a mouse OB GCL injected with rAAV-Cre. Scale bar, 250 μm. (**b**) Western blot of whole-olfactory bulb homogenates using the β3-subunit antibody in rAAV-Cre-injected versus non-injected *GABRB3*^*loxP/loxP*^ mice (each six samples from three mice). The β3-subunit appears as a band of 55 kDa, consistent with its predicted molecular weight. Densitometric analysis (mean±s.e.m.) revealed a significant difference (*t*-test; *P=0.016). (**c**) Number of DAPI-positive, Cre-positive and NeuN-positive cells in the GCL infected mice with AAV-Cre (mean±s.e.m.). Top: the colocalization of Cre-positive nuclei and DAPI was 42±2% (*n*=3 mice). Bottom: the colocalization of Cre- and NeuN-positive nuclei was 68±2% (*n*=3 mice). Images show a projection of 10 confocal image frames covering an axial distance of 1.5 μm. Scale bar, 50 μm.

To abolish β3-subunit expression in GCs, we took advantage of a mouse line harbouring loxP sites flanking the exon 3 of the *GABRB3* gene^29^. Specific deletion of the β3-subunit in GCs was achieved by stereotaxic delivery of rAAV-Cre particles into the GCL. Immunohistochemistry against Cre-recombinase revealed that infected cells were almost exclusively present in the GCL sparing the MCL and other OB layers (Fig. 3a). Western blot analysis showed a significant decrease in the β3-subunit in the OB (Fig. 3b, *n*=3 mice in each group, two samples per mouse, *t*-test, *P*=0.016). To probe the efficiency of GC infection, we determined the colocalization of Cre-recombinase (all infected cells), 4,6-diamidino-2-phenylindole (DAPI; nuclei of all cell types) and NeuN (neurons; Fig. 3c). DAPI and NeuN were colocalized in 68±2% (*n*=3 mice) of the cells, approximately reflecting the ratio of neurons and glia. DAPI and Cre were colocalized in 42±2%, while NeuN and Cre-recombinase overlapped in 63±3% of the cells, suggesting that β3-deletion occurred in ∼63% of the GCs.

**Figure 4:**
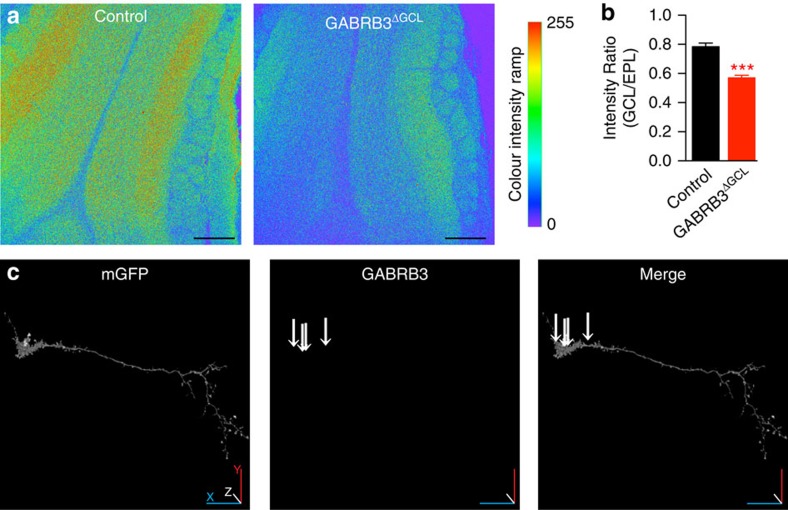
Deletion of β3-subunits in GCL. (**a**) Single confocal planes showing examples of GABRB3 immunosignals in the OB of control and GABRB3^ΔGCL^ mice 3 weeks after stereotaxic injection. Scale bars, 250 μm. (**b**) The ratio of intensities measured within the GCL and ECL of images such as shown in **a** (mean±s.e.m.). The average fluorescence intensity of seven regions of interest (100 μm × 100 μm) each in the GCL and EPL, placed on opposite sides of the MCL and facing each other, were determined for each section using ImageJ. The averaged ratios were calculated from sections obtained from three control (0.78±0.03) and four GABRB3^ΔGCL^ mice (0.57±0.02; *t*-test; ***P=0.0005). (**c**) Example of a GC in a GABRB3^lox/lox^ mouse expressing Cre recombinase and mGFP as a marker (further examples are shown in Supplementary Fig. 4B). Only a small number of faint β3-immunosignals (marked with arrows) are detectable 3 weeks after viral perturbation (representative for three GABRB3^lox/lox^ mice co-injected with rAAV-Cre (1:1,000 diluted in 1 × PBS) and rAAV-DIO-mGFP (mixed in a 1:1 ratio with diluted rAAV-Cre). Cartesian axes *x*(blue), *y* (red) and *z* (white), image size 235 μm × 235 μm, pixel size=0.229 μm, 90 image frames covering a depth of 13.3 μm.

The deletion of the β3-subunit in GCs was further examined by β3-immunostaining of GABARB3^lox/lox^ and control tissue 3 weeks after injecting rAAV-Cre designed to yield a dense expression of Cre in a large fraction of GCs (Supplementary Fig. 4A). Immunostaining with β3-antibody (Fig. 4a) was quantified in the GCL and ECL, and an expression ratio was calculated (Fig. 4b). The ratio approach was chosen to normalize β3-expression in the GCL to that in the EPL because most of the β3 signals in the EPL originate from MCs and thereby serve as an internal control. The decrease in the expression ratio (Fig. 4b) confirms a reduction of the β3-subunit in the GCL. The extent of the ratio decrease is consistent with the overall efficiency of viral infection (see Fig. 3). To further assess changes in β3-expression on the level of individual GCs, rAAV-Cre (diluted 1:1,000) was co-expressed with rAAV-DIO-mGFP, thereby producing a β3-subunit knockout in individually resolvable GCs. We found a strongly reduced expression 21 days after virus injection (Fig. 4c and Supplementary Fig. 4B). This time course is consistent with our previous experience in deleting ionotropic glutamate receptor subunits^19^. Furthermore, this approach provided additional evidence for the specificity of the antibody used against the β3-subunit and the 3D immunohistochemistry approach.

In summary, these results show that our strategy is suitable to achieve an efficient reduction in β3-subunit expression within the GCL not affecting neurons of other OB layers. Although the vast majority of cells in the GCL are GCs, neurons other than GCs^22^,^32^ may get transduced with AAV-Cre particles. Hence, rAAV-Cre-infected mice are referred to as GABRB3^ΔGCL^.

### β3-deletion in GCs reduces GABA_A_R-mediated mIPSCs

**Figure 5:**
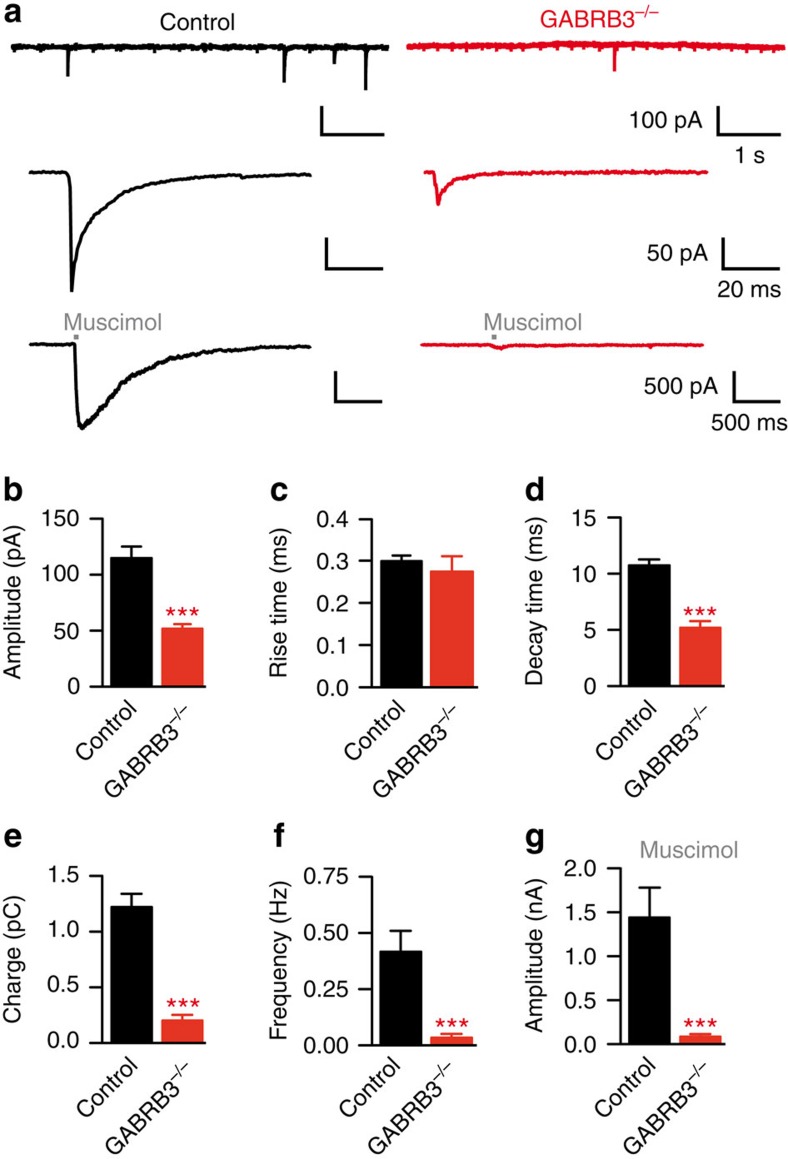
GABRB3 deletion in GCs reduces GABA_A_-receptor-mediated currents. (**a**) Representative recordings of spontaneous activity in control (upper panel, black) versus GABRB3^−/−^ (upper panel, red) GCs in the presence of 50 μm APV, 10 μm CNQX and 1 μm TTX. Central panels: average mIPSC from 58 (control) and 3 (GABRB3^−/−^) events recorded in a 2-min session. Lower panels: local application of 100-μm muscimol for 50 ms. Representative examples taken from 8 recordings in control (black) and 14 recordings in knockout cells (red). (**b**) mIPSC amplitudes in control and knockout cells (control 115±10 pA, *n*=14; GABRB3^−/−^: 51±3 pA, *n*=6; *t*-test; ***P<0.001; mean±s.e.m.). Six out of twelve GABRB3^−/−^ cells had at least one event. (**c**) mIPSC rise time (control: 0.3±0.02 ms, *n*=14; GABRB3^−/−^: 0.27±0.05 ms, *n*=6; mean±s.e.m.). (**d**) mIPSC decay time constant (control: 10.8±0.5 ms, *n*=14; GABRB3^−/−^: 5.2±0.6 ms, *n*=6; *t*-test; ***P<0.001; mean±s.e.m.). (**e**) mIPSC charge (control: 1.2±0.12 pC, *n*=14; GABRB3^−/−^: 0.2±0.05 pC, *n*=6; *t*-test; ***P<0.001; mean±s.e.m.). (**f**) mIPSC frequency (control: 0.4±0.09 Hz, *n*=14; GABRB3^−/−^: 0.035±0.02 Hz, *n*=12; *t*-test; ***P<0.001; mean±s.e.m.). (**g**) IPSC amplitude on local application of 100 μM muscimol (control: 1.4±0.4 nA, *n*=8; GABRB3^−/−^: 0.08±0.03 nA, *n*=14; *t*-test; ***P<0.001; mean±s.e.m.).

Deletion of the β3-subunit, a member of the essential β-subunits^25^, has been shown to abrogate synaptic GABA_A_R-mediated currents^33^ in GCs. To confirm this result, *in vitro* whole-cell voltage-clamp recordings were performed in acute OB slices from GCs expressing GFP and Cre-recombinase (knockout cells) and from non-labelled GCs (control cells). Miniature inhibitory post-synaptic currents (mIPSCs) were almost completely abolished in GCs lacking the β3-subunit (Fig. 5a, upper panels *n*=14 control cells and *n*=12 knockout cells). Indeed, only 6 out of 12 Cre-positive cells had at least one mIPSP, although the amplitude of these events was strongly reduced in comparison with control cells (Fig. 5b; *n*=14 control cells and *n*=12 knockout cells; *t*-test; *P*<0.001). Although the remaining events did not differ in the rise time (Fig. 5c; *n*=14 control cells and *n*=12 knockout cells; *t*-test; *P*=0.56), the decay time was significantly decreased (Fig. 5d; *n*=14 control cells and *n*=12 knockout cells; *t*-test; *P*<0.001) and the charge of the remaining mIPSCs reduced (Fig. 5e; *n*=14 control cells and *n*=12 knockout cells; *t*-test; *P*<0.001). Control cells exhibited a low mIPSC frequency and, on deletion of the β3-subunit, mIPSCs occurred only rarely (Fig. 5f; *n*=14 control cells and *n*=12 knockout cells; *t*-test; *P*=0.001). Furthermore, local application of 100-μM muscimol using a 50-ms puff (Fig. 5a, lower panels) revealed a strong reduction in the GABA_A_R current in knockout cells (Fig. 5g; *n*=8 control cells and *n*=14 knockout cells; *t*-test; *P*<0.0001). This is consistent with the observed strong reduction in the number of β3-positive clusters (see above) and previously published results obtained in global β3-subunit knockout mice^7^. The remaining clusters had much smaller intensities and therefore contained a strongly reduced number of β3-subunits. The latter will cause a decrease in the mIPSC amplitudes towards the noise level of the recordings, giving rise to an apparent reduction in mIPSC frequency. Variability in receptor turnover, viral infection rate and incubation time after viral injection may explain that GABA_A_R-mediated currents were abolished in only 50% of the cells, while the remaining cells showed small currents. In conclusion, these results provide evidence for a strong decrease in functional GABA_A_-Rs at inhibitory input synapses of GCs in GABRB3^ΔGCL^ mice.

### Phasic inhibition of GCs controls strength of MC inhibition

**Figure 6:**
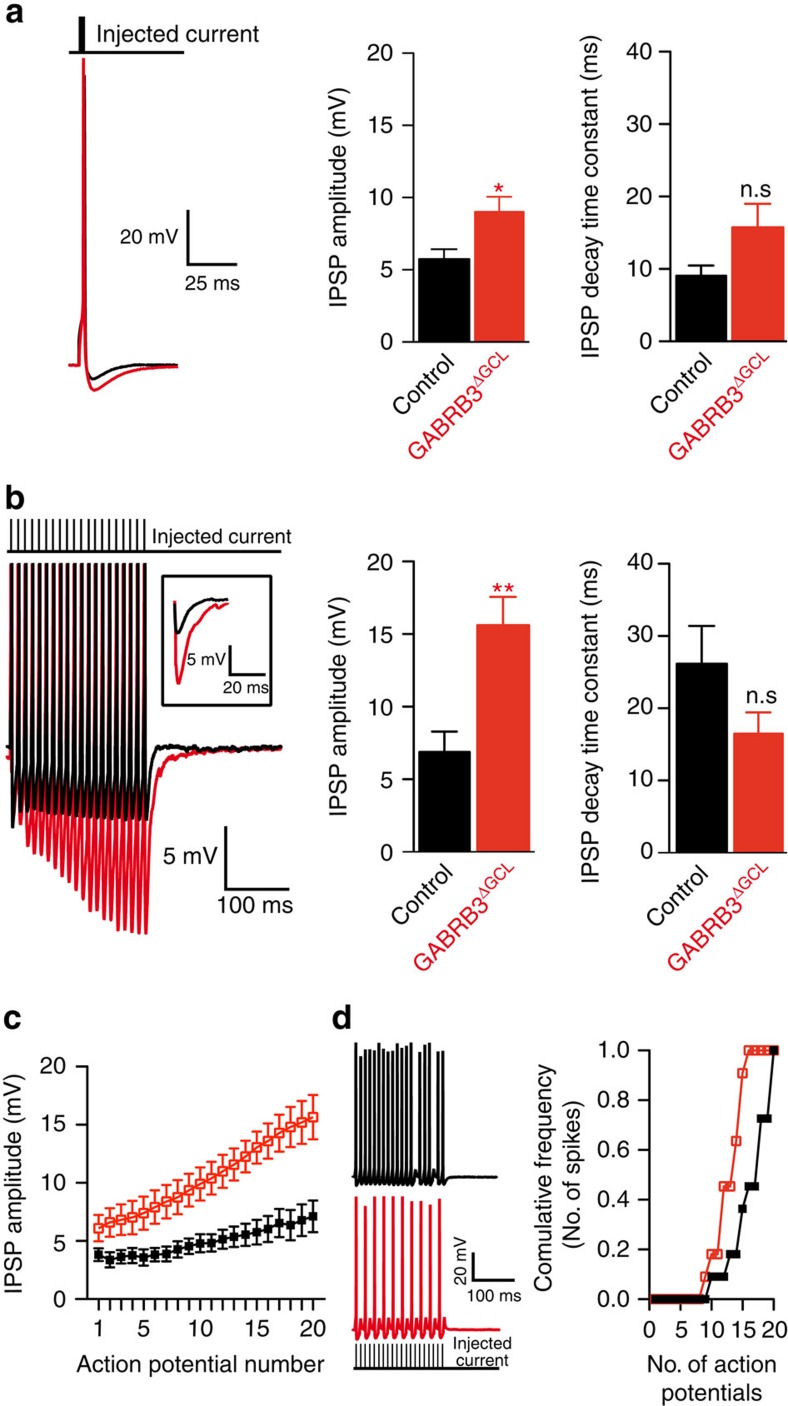
GCs inhibition controls the strength of MC inhibition. (**a**) Single-pulse current injection into a MC (control cells: 445±65 pA, *n*=11; knockout cells: 441±56 pA, *n*=11; *P*=0.96). Left: representative example of control (black) and knockout (red) cells. Centre: The mean IPSC amplitude±s.e.m. (control 5.8±0.7 mV, *n*=11 cells; GABRB3^ΔGCL^: 9±1 mV, *n*=11 cells; *t*-test; *P=0.017). Right: decay time constant (control: 9±1.4 ms, GABRB3^ΔGCL^: 15.8±3.3 ms; *t*-test; *P*=0.07; mean±s.e.m.). (**b**) Twenty suprathreshold current injections at a frequency of 100 Hz (left), IPSP amplitudes in control and GABRB3^ΔGCL^ mice (control: 6.9±1.4 mV, *n*=11 cells; GABRB3^ΔGCL^: 15.7±1.9 mV, *n*=11 cells; *t*-test; **P=0.0014; mean±s.e.m.; centre), decay time constant (control 26±5.2 ms, *n*=11 cells; GABRB3^ΔGCL^ 16.5±3 ms, *n*=11 cells; *t*-test; *P*=0.12; mean±s.e.m.; right). Measurements are from the same cells as in **a**. (**c**) Dependency of IPSP amplitude (mean±s.e.m.) on stimulus number during the 100-Hz train. (**d**) AP failures in MCs of control and GABRB3^ΔGCL^ mice. Left: representative AP trains for control (black) and GABRB3^ΔGCL^ (red). Right: cumulative frequency of APs fired during a series of 20 APs (*n*=10 control cells and *n*=11 GABRB3^ΔGCL^ cells; Cumulative-Gaussian *F*-test, *P*<0.0001).

We next aimed at evaluating the physiological consequences of β3-subunit abrogation in GCs on recurrent inhibition of MCs. To accomplish this, whole-cell current-clamp recordings were established from MCs in 300-μm-thick acute horizontal OB sections at 34 °C. Single-current injections with increasing amplitude (see Methods) were applied until MCs fired an action potential (AP). Immediately after AP firing, a strong hyperpolarization of the MC membrane potential was observed, reflecting recurrent inhibition of the MC (Fig. 6a). The amplitude of the inhibitory post-synaptic potential (IPSP) was increased in MCs recorded in GABRB3^ΔGCL^ mice compared with control (Fig. 6a; *t*-test; *P*=0.017; *n*=11), while the IPSP decay time constant was not significantly changed (Fig. 6a; *t*-test; *P*=0.07; *n*=11). Thus, recurrent inhibition elicited by a single AP in MCs was stronger in the absence of GABA_A_R in the majority of surrounding GCs.

MCs have been shown to respond to odour stimuli with brief bursts of APs^12^. To test frequency dependence of recurrent inhibition, 20 consecutive APs were elicited by applying suprathreshold currents (control cells: 654.5±74.3 pA, *n*=11, GABRB3^ΔGCL^: 809.1±63.9 pA, *n*=11; *t*-test; *P*=0.13). The IPSP amplitude at the end of the train was greatly increased in MCs of GABRB3^ΔGCL^ mice (Fig. 6b; *t*-test; *P*=0.001; *n*=11). The IPSP decay time constant was not significantly changed (Fig. 6b; *t*-test; *P*=0.12; *n*=11). When compared with single AP firing in controls, the decay time constant was significantly increased (*t*-test; *P*=0.005; *n*=11), while in MCs of GABRB3^ΔGCL^ mice it remained unchanged (*t*-test; *P*=0.87; *n*=11). Furthermore, we analysed the IPSP amplitudes during the train of APs (Fig. 6c). We found only a small increase in MCs of control mice while the IPSP amplitude strongly increased in MCs of GABRB3^ΔGCL^ mice (two-way analysis of variance (ANOVA); *P*=0.0017; *n*=11). Moreover, using the same stimulation thresholds we found that control MCs showed fewer failures in AP generation compared with MCs from GABRB3^ΔGCL^ mice (Fig. 6d; Cumulative-Gaussian *F*-test, *P*<0.0001; *n*=10 control cells and *n*=11 GABRB3^ΔGCL^ cells). These observations strongly suggest that GC inhibition is an important mechanism regulating MC firing properties and tuning the amount of inhibition that MCs can recruit on stimulation.

### β3-deletion in the GCL accelerates odour discrimination

**Figure 7:**
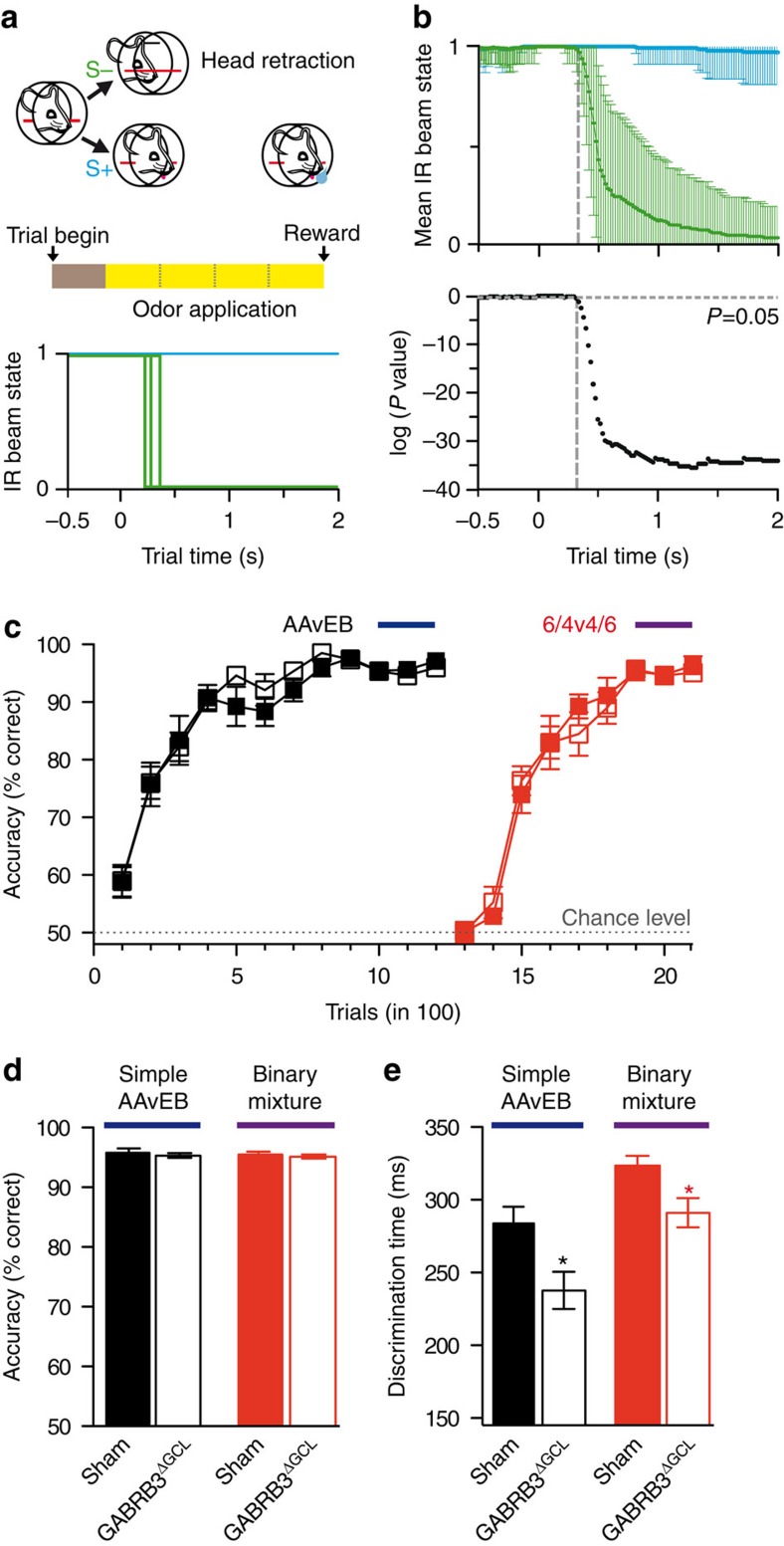
Accelerated odour discrimination times in GABRB3^ΔGCL^ mice. (**a**) Scheme of the go/no-go paradigm. Pictograms show mouse head in the odour port with the infrared light beam shown in red. Unrewarded (S−, green) odour results in head retraction, rewarded odour (S+, blue, same colour scheme used in remaining panels) in licking behaviour. The lower panel shows abrupt changes of the light beam on head removal for three examples of individual trials. (**b**) ODT calculation. Representative data set from a control mouse trained to discriminate binary mixtures (ODT=324 ms; vertical grey line). Each curve consists of 125 time bins (mean±s.d.). S+ and S− trials are compared by bootstrapping statistics, defining the ODT as the time bin at which the two curves are significantly different. (**c**) Learning curves for the discrimination of dissimilar odourants AA and EB, and their binary 60:40 mixture (mean±s.e.m.). Coloured bars (monomolecular odours in dark blue and binary mixtures in purple) depict the trials used to calculate odour discrimination time. Both groups of mice (*n*=8 in each group) learned equally well (ANOVA; simple: *F*=1.307, *P*=0.255; binary mixture: *F*=0.089, *P*=0.765). (**d**) Odour discrimination accuracy. Both groups of mice performed equally well after learning to discriminate between simple odours (control: 95.8±0.7%, *n*=8; GABRB3^ΔGCL^: 95±0.4%, *n*=8; *t*-test; *P*=0.609; mean±s.e.m.) and its binary mixtures (control: 95.5±0.5%, *n*=8; GABRB3^ΔGCL^: 95.2±0.3%, *n*=8; *t*-test; *P*=0.602; mean±s.e.m.). (**e**) Odour discrimination time. GABRB3^ΔGCL^ mice were faster discriminating simple odours (control: 284±11 ms, *n*=8; GABRB3^ΔGCL^: 238±13 ms, *n*=8; *t*-test; *P=0.016; mean±s.e.m.) and binary mixtures (control: 324±7 ms, *n*=8; GABRB3^ΔGCL^: 291±10 ms, *n*=8; *t*-test; *P=0.018; mean±s.e.m.).

The observation that recurrent inhibition of MCs is strongly increased in GABRB3^ΔGCL^ mice and that increased inhibition accelerates odour discrimination^19^ leads us to hypothesize that mice lacking the β3-subunit in GCs would require less time to discriminate odours. To test this, we used a go/no-go operant conditioning odour discrimination paradigm^34^ illustrated in Fig. 7a. Briefly, a trial is self-initiated by the mouse inserting its head into the odour port and thereby breaking the infrared light beam. On odour application the mouse retracts its head when an unrewarded (S−) odour is applied or stays in the odour port and starts licking until the water reward is applied at the end of the trial. The corresponding state of the infrared light beam is shown in the lower panel for three individual example trials. After sampling hundreds of trials, a mean fraction of the beam being disrupted at any given time point can be calculated and a bootstrapping statistical procedure be used to determine the time point at which the S+ and S− curves differ significantly, defined as the ODT (Fig. 7b, see Methods for a more detailed description). Mice require ∼1,000 trials to achieve >90% accurate discriminations; we refer to this learning process as odour discrimination learning. ODT is determined only after a stable performance at >90% correct identifications has been reached.

In the first experiment we applied our previously established paradigm^19^. The OBs of mice were stereotaxically injected with rAAV-eGFP (sham group, *n*=8) or with rAAV-Cre (GABRB3^ΔGCL^ group, *n*=8). First, mice were trained to discriminate between the monomolecular odourants amyl acetate (AA) and ethyl butyrate (EB). Both sets of mice readily acquired the discrimination task and reached a discrimination accuracy exceeding 90% within ∼1,000 trials (Fig. 7c). Second, a 60/40% binary mixture of these odourants was used to generate highly similar stimuli. Discrimination accuracy initially dropped to a chance level but increased to >90% after ∼600 trials (Fig. 7c). The learning curves of control and GABRB3^ΔGCL^ knockout mice were indistinguishable for both monomolecular odourant discrimination and binary mixture discrimination (Fig. 7c,d; two-way ANOVA; *P*=0.29 for simple odours and *P*=0.77 for binary mixtures; *n*=8). ODTs were measured when performance exceeded 90% (Fig. 7d). We found that monomolecular odourant discrimination and mixture discrimination were both accelerated in mice lacking the β3-subunit in GCs (Fig. 7e; *t*-test; monomolecular odourants *P*=0.016; binary mixtures *P*=0.018; *n*=8). Thus, in contrast to previous molecular perturbations affecting MC inhibition, abrogating GC inhibition resulted in accelerated discrimination independent of the similarity of odour representations in the OB.

**Figure 8:**
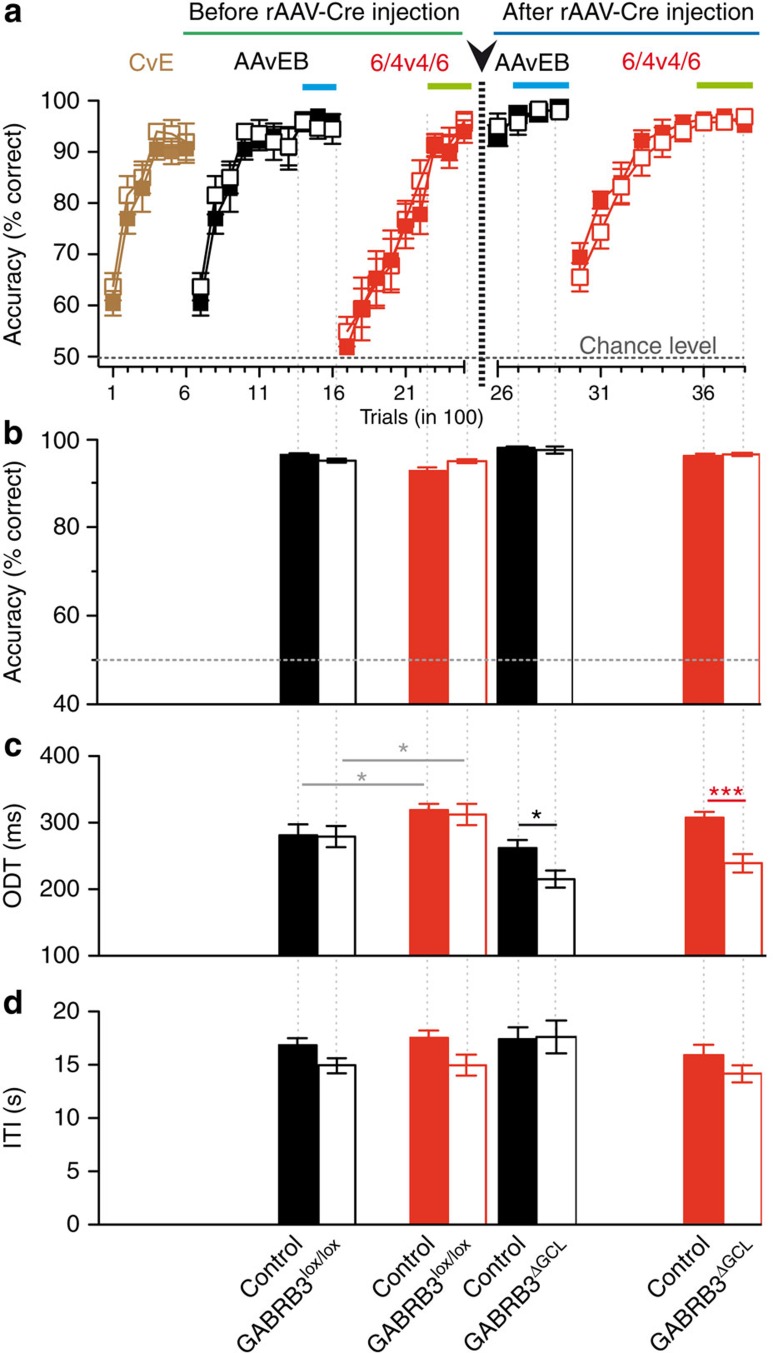
Accelerated odour discrimination times revealed in a longitudinal paradigm. (**a**) Learning curves. Cineol and eugenol (CvE) were used for task habituation. Test odours, AA and EB (AAvEB), and their binary mixture in a 60:40 ratio (6/4v4/6) were used to measure the discrimination times before and after rAAV-Cre injection (mean±s.e.m.). Control mice (*n*=9) and GABRB3^lox/lox^ (*n*=9) neither differed in learning before (ANOVA; CvE: *F*=3.084, *P*=0.08; AAvEB: *F*=0.655, *P*=0.420; 6v4: *F*=0.523, *P*=0.471) nor after rAAV-Cre injection (AAvEB: *F*=0.004, *P*=0.95; 6v4: *F*=3.208, *P*=0.076). The coloured bars (simple odours: light blue; binary mixtures: light green) depict the trials used for the quantifications in **b**–**d**. (**b**) Odour discrimination accuracy (mean±s.e.m.) was not significantly different for simple odours (control: 96.3±0.3%, *n*=9; GABRB3^lox/lox^: 95±0.4%, *n*=9; ANOVA; *P*=0.077) or binary mixtures (control: 93±1.41%, *n*=9; GABRB3^lox/lox^: 95±0.4%, *n*=9; ANOVA; *P*=0.313). After rAAV-Cre injection, both groups of mice continued to perform equally well for simple odours (control: 97.8±0.35%, *n*=9; GABRB3^ΔGCL^: 97±0.8%, *n*=9; ANOVA; *P*=0.593) and for the binary mixtures (control: 96±0.5%, *n*=9; GABRB3^ΔGCL^: 96±0.35%, *n*=9; ANOVA; *P*=0.945). (**c**) Odour discrimination times. Before rAAV-Cre injection, control (*n*=9) and GABRB3^lox/lox^ (*n*=9) did not differ in ODTs (mean±s.e.m.) of simple odours (control: 291±11 ms; GABRB3^lox/lox^ 280±16 ms; ANOVA; *F*=1.614; *P*=0.17) and binary mixtures (control: 320±9 ms; GABRB3^lox/lox^: 313±16 ms; ANOVA; *F*=0.979; *P*=0.52); however, the ODTs between simple odours and binary mixtures differed significantly (control: *P=0.038; GABRB3^lox/lox^: *P=0.047; *t*-test). After rAAV-Cre injection, GABRB3^ΔGCL^ had faster ODTs of simple odours (control: 263±12 ms; GABRB3^ΔGCL^ 216±13 ms) and binary mixtures (control 306±9 ms; GABRB3^ΔGCL^ 237±14 ms; ANOVA, simple odours: *F*=4.654, *P=0.047; binary mixtures: *F*=9.212, ***P=0.008). (**d**) ITI as an indicator of motivation. Before rAAV-Cre injection, control mice (simple odours: 19±2 s; binary mixtures: 17±0.7 s; *n*=9) and GABRB3^lox/lox^ mice (simple odours: 15±0.7 s; binary mixtures: 15±0.99 s; *n*=9) do not differ significantly in the ITI (mean±s.e.m.; ANOVA; F=1.93; *P*=0.18). After Cre injection, control mice (simple odours: 18±1 s; binary mixtures: 16±1 s; *n*=9) and GABRB3^ΔGCL^ mice (simple odours: 17.6±1.6 s; binary mixtures: 14.2±0.8 s; *n*=9) also did not differ significantly in the ITI (mean±s.e.m.; ANOVA; *F*=3.621; *P*=0.078).

In the second experiment we aimed at further controlling for unspecific effects of surgery, Cre-recombinase expression and other potentially confounding variability factors. To achieve this, we applied a longitudinal design consisting of sessions including discrimination in non-injected wild-type (*n*=9) and GABRB3^lox/lox^ (*n*=9) mice followed by viral perturbation and subsequent sessions comparing control and GABRB3^ΔGCL^ mice. This allowed us to follow behavioural parameters longitudinally in every single mouse. For task habituation, mice were first trained to discriminate the simple odours cineol (C) and eugenol (E) until accuracy exceeded 80%. Subsequently, mice rapidly learned to discriminate monomolecular odourants AA/EB and highly similar binary mixtures thereof with high accuracies (Fig. 8a). The learning curves of the two groups (Fig. 8a; two-way ANOVA; simple odours *P*=0.4195 and binary mixtures *P*=0.4709; *n*=9) and steady-state discrimination accuracy were indistinguishable (Fig. 8b; two-way ANOVA; simple odours *P*=0.42 and binary mixtures *P*=0.47; *n*=9). ODTs determined from the last 300 trials were indistinguishable between the groups, measuring ∼285 and 317 ms for monomolecular odourants and binary mixtures, respectively (Fig. 8c). The increased discrimination time for binary mixtures is consistent with our previous results^19^,^34^.

Next, all mice were stereotaxically injected with rAAV-cre (Fig. 8, arrowhead and vertical dashed line), and after recovery from surgery the same cohort of mice performed a second session of behaviour using the same test odour pairs. Discrimination of AA versus EB started at high discrimination accuracy consistent with the mice remembering the odour pair from previous training sessions and reached saturated performance already after less than 200 trials (Fig. 8a,b). Binary mixture discrimination started at 65% accuracy and reached saturating accuracy after 600 trials, suggesting that mice also remembered this odour discrimination, although not as efficiently as the monomolecular odourant discrimination (Fig. 8b). The learning curves and discrimination accuracies were indistinguishable between the two groups (Fig. 8a; two-way ANOVA; *P*=0.95 for simple odours and *P*=0.08 for binary mixtures; *n*=9). The ODTs for monomolecular odourants and binary mixtures were shorter in this compared with the pre-injection session, possibly because of continued training (two-way ANOVA; *P*=0.0015 for simple odours and *P*=0.0024 for binary mixtures; *n*=9). However, GABRB3^ΔGCL^ mice revealed significantly faster ODTs of both monomolecular odourants as well as binary mixtures (Fig. 8c; two-way ANOVA; simple odours *P*=0.046 and binary mixtures *P*=0.008; *n*=9), confirming the results of the first discrimination experiment. The motivation of mice to perform the task was controlled over the entire experiment by measuring the inter-trail interval (ITI). No changes were observed either before or after the surgery (Fig. 8d; two-way ANOVA; simple odours *P*=0.083 and binary mixtures *P*=0.11; *n*=9) and neither between groups (two-way ANOVA; simple odours *P*=0.183 and binary mixtures *P*=0.078; *n*=9).

**Figure 9:**
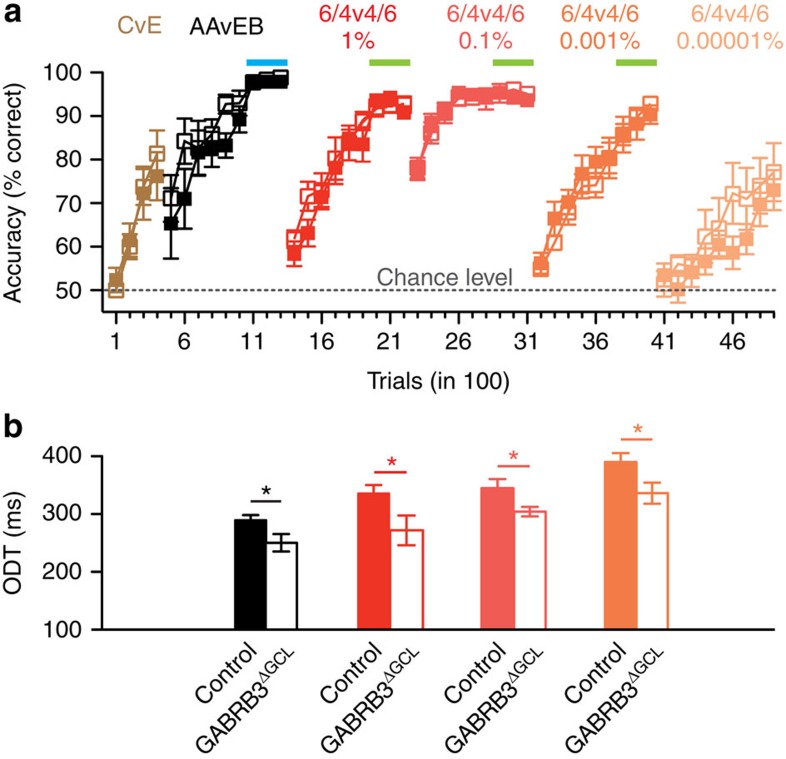
ODT is faster in GABRB3^ΔGCL^ mice when challenged with more difficult discrimination tasks. (**a**) Learning curves. Cineol and eugenol (CvE) were first used for task habituation. Afterwards, test odours, AA and EB (AAvEB) and their binary mixture in a 60:40 ratio (6/4v4/6) were used in progressively smaller concentrations to generate discrimination tasks of increasing difficulty. No differences in learning performance were observed between control (*n*=8) and GABRB3^ΔGCL^ (*n*=8) mice for the different odour concentrations tested (mean±s.e.m.; ANOVA; *P*>0.05). The coloured bars (simple odours light blue and binary mixtures light green) depict the trials used for the determination of odour discrimination times. Because mice were unable to reach a high performance level (>90%) in the last dilution tested, the discrimination time for this concentration was not determined. (**b**) Odour discrimination times of the stimuli tested in **a**. All GABRB3^ΔGCL^ (*n*=8) mice exhibited significantly faster discrimination times than control (*n*=8) mice. Simple odours: control 289±9 ms, GABRB3^ΔGCL^ 250±15 ms, *P<0.05; 1% 6/4v4/6: control 335±9 ms, GABRB3^ΔGCL^ 272±26 ms, *P=0.05; 0.1% 6/4v4/6: control 345±16 ms, GABRB3^ΔGCL^ 304±8 ms, *P<0.05; 0.001% 6/4v4/6: control 390.0±16 ms, GABRB3^ΔGCL^ 336±18 ms, *P<0.05; mean±s.e.m. All pairs of data sets were tested for significance using the *t*-test.

Our conclusions thus far rest on using two monomolecular odourants and their binary 60/40 mixtures; however, accelerated odour discrimination may be confounded by ceiling effects and may not apply to stimuli of different similarities. To test this, we first trained a new batch of mice (*n*=8 controls, *n*=8 GABRB3^ΔGCL^) to discriminate increasing dilutions of the binary mixtures (Fig. 9). After pretraining with cineol versus eugenol, mice rapidly learned to discriminate monomolecular odourants AA and EB. After learning binary mixture discrimination at our standard odour concentration of 1%, they equally well learned to discriminate 0.1% of the mixture. However, starting with a dilution of 0.001%, the learning curve was less steep and required longer to reach our target criterion of >90% discrimination accuracy. At 10^−5^%, mice performed at only 75% accuracy even after 900 trials, suggesting that even under non-saturated behavioural performance the difference in discrimination time persisted. The learning curves and discrimination accuracies did not differ between control and GABRB3^ΔGCL^ mice while ODTs were significantly reduced in all situations (Fig. 9). These results indicate that increased inhibition accelerates odour discrimination independent of the similarity and difficulty of the discrimination task. To further corroborate this result, we tested discrimination of carvone stereoisomers and cineol versus eugenol, both monomolecular odourants and their binary mixtures (Supplementary Fig. 5). Learning the discrimination of carvone and cineol versus eugenol mixtures took more time while 90% discrimination accuracy could be reached. However, ODTs were reduced in GABRB3^ΔGCL^ mice in all situations, further supporting the prediction that the accelerated odour discrimination is a general feature of increased MC inhibition.

In conclusion, increased MC inhibition, caused by disinhibition of GCs, accelerates odour discrimination independent of odour similarity.

## Discussion

In this study we investigated the cellular and behavioural role of GC inhibition. In contrast to previous studies examining a global β3-subunit knockout^7^, we deleted the *GABRB3* gene^29^ selectively in the GCL. This ‘genetic' disinhibition of GCs increased the inhibition of MCs and accelerated odour discrimination. ODT was decreased independently of the similarity of odourant representations in the OB. These observations suggest that GCs are inhibited by a persistent GABAergic input and/or an odour-evoked feedforward signal. This inhibitory drive may serve as a mechanism to regulate the strength of MC inhibition and thereby adjust the time required to discriminate odours.

Our study provides the second example of a targeted molecular GC perturbation, resulting in a gain-of-function phenotype in odour discrimination. Similar to our previous study deleting GluA2 in the GCL^19^, we found increased MC inhibition and accelerated discrimination after deleting β3-subunits. Hence, with two different molecular perturbations converging on the same cellular and behavioural outcome, we suggest that increased MC inhibition, in general, decreases ODT while reduced MC inhibition increases ODT^19^. Therefore, the relative strength of GC inhibition fine-tunes MC inhibition and ODT. The level of inhibition could affect gamma cycle frequency and with it the speed of transmitting a certain number of synchronous MC APs towards higher brain areas.

Why would the OB generate changes in ODT rather than cortical areas rendering discrimination decisions? We suggest that a certain level of MC inhibition is required to produce accurate odour discrimination decisions. Strong MC inhibition will reach this level of inhibition faster than weak MC inhibition (see Fig. 8b, Abraham *et al*.^19^; this Figure shows that the time course of inhibition matches the differences in ODT observed for molecular perturbations). Therefore, the time course of inhibition build-up in MCs may define a substantial fraction of the ODT. Downstream brain areas directly concerned with decision-making may contribute to ODT too; however, these contributions are predicted to work on a shorter timescale.

Our results show a prominent link between MC inhibition and ODT; yet, odour discrimination learning appears to be unaffected by the strength of MC inhibition. This lack of an effect could reflect limitations in the odourant space sampled with our behavioural assay and the difficulty of the discriminations assessed. However, we think that this is unlikely because we used a wide range of odourants, binary mixtures and dilutions. Furthermore, forebrain-specific deletion of GluA2 accelerated discrimination learning while deletion in the OB did not^35^. These results suggest that discrimination learning may primarily occur in cortical olfactory areas; yet, effects of OB inhibition may become evident when pushing the difficulty of discrimination tasks further.

Similar olfactory stimuli generate similar spatiotemporal representations^34^,^36^ that in turn require different amounts of processing time in the OB network to achieve pattern separation. In contrast to the expectation that discrimination of dissimilar stimuli may work without inhibition of MCs, we show that this is not the case. Consistently, a tendency for accelerated ODT was also found after deletion of GluA2 receptors in GCs (see Fig. 5d in ref. 19).

Our colocalization analysis of VIAAT, gephyrin and β3-subunit clusters in GCs demonstrates that GABA_A_Rs in GCs are located synaptically and hence mediate phasic rather than tonic inhibition of GCs. This conclusion is inconsistent with the proposal that tonic, extrasynaptic inhibition dominates over phasic, synaptic inhibition in GCs^36^. Their conclusion was based on using furosemide as an antagonist of GABA_A_Rs containing the β3- and α4- or α6-subunits (the latter not expressed in GCs^25^), independent of the presence of γ2- or δ-subunits^37^,^38^. Because we have demonstrated a synaptic distribution of the β3-subunit in GCs, extrasynaptic receptors would have to be homomeric α4-channels. However, such GABA channels have not yet been described to exist *in vivo*^39^,^40^. The findings of Labarrera *et al*.^36^ may be explained with the inhibition of KCC2 (ref. 41) by furosemide, resulting in decreased GABAergic inhibition due to accumuated intracellular chloride and increased AP firing (see Fig. 4 (ref. 36)). Alternatively, our imaging approach may lack the resolution and sensitivity to detect single GABA_A_R channels distributed at a low density across the plasma membrane of GCs. Hence, a tonic component in addition to phasic inhibition may exist^25^,^40^; however, its dominant impact^36^ seems highly unlikely according to our results.

Several cell types known to exist in the OB^42^ may provide inhibitory input to GCs, for example short-axon cells or Blanes cells. The latter were shown by Pressler and Strowbrige^22^ to fire persistently on glomerular stimulation and to induce a GABAergic synaptic current in GCs. Hence, MC APs may spread via collateral axons and activate Blanes cells that in turn inhibit GCs. Depending on the connectivity of the Blanes cells, a single such cell could potentially inhibit a large number of GCs and thereby decrease MC inhibition in a manner precisely tuned to the onset of the odour stimulus. Such a circuit predicts that a MC may even disinhibit itself, assuming appropriate wiring, a function that could contribute to contrast enhancement. In conclusion, our physiological and behavioural findings are consistent with an inhibitory drive on GCs as suggested by Pressler and Strowbridge^22^. Yet, we cannot exclude that GCs are also inhibited by other cells of the OB or by long-range GABAergic neurons projecting from areas outside the OB, similar to ChAT- and GAD-positive neurons in the horizontal limb of the diagonal band^43^.

Our electrophysiology data suggest that sparse connectivity between GCs and MCs^44^,^45^ might be a consequence of GC inhibition. This is consistent with the observation that a single MC AP elicits a similar degree of inhibition compared with 20 consecutive APs in the unperturbed OB (see Fig. 6a,b, compare control IPSP amplitudes). In contrast, when inhibition of GCs is nearly abolished after β3-subunit deletion, MC inhibition increases strongly with the number of APs, indicating that the mechanism of inhibiting GCs determines the frequency-dependent strength of inhibition of MCs (Fig. 6c).

GC excitatory inputs originating from MC dendrites may coincide with inhibitory inputs, a mechanism proposed to suppress multiple spiking of GCs^46^. We observed that in wild type, the decay time of the IPSP after 20 consecutive APs increases about four times when compared with that elicited by a single AP, while after β3-subunit deletion this relationship is lost (Fig. 6a,b, compare decay time constants). This observation supports the hypothesis that GC inhibition might be an important mechanism to define the kinetics of MC inhibition. Strong hyperpolarization of the GC may trigger a long-lasting T-type Ca^2+^ current^11^ that may prolong GABA release from GC gemmules.

In this study we established a longitudinal behavioural design that allows comparisons of individual mice before and after deletion of the target gene. We found that the overall variability of the derived parameters is smaller and that a larger number of controls can be achieved in one experiment. By using a second set of wild-type mice, the line harbouring the floxed allele can be directly compared with wild type and the effect of the injected virus construct on wild type can be assessed.

## Methods

### Animals

In all experiments, mice were housed in standard mouse cages in a constant day–night cycle (12–12 h) and in a temperature- and humidity-controlled environment. Mice having the exon 3 of the *GABRB3* gene flanked by loxP sites (GABRB3^lox/lox^) were purchased from Jackson Laboratories (B6;129-Gabrb3^tm2.1Geh^/J [Jax:008310]) and backcrossed multiple generations with C57Bl/6 wild-type mice purchased from Charles River. Genotyping was performed according to Jackson Laboratories specifications. Mice were handled in agreement with the European FELASA guidelines, and all procedures were approved of by the authorities (Regierungspräsidium Karlsruhe).

### Acute targeted genetic perturbation

Male, 21-day-old mice were stereotaxically injected using a BENCHMARK stereotax. The rAVV chimeric vectors (1:1 ratio of AVV1 and AAV2 capsid proteins) were injected in the GC layer of the OB, as previously described^19^. Briefly, the rAAV-Cre vector contained the coding sequence for Cre recombinase, N terminally fused to the haemagglutinin-tag and the SV40 nuclear localization signal in a rAAV plasmid backbone (pAM) containing the 1.1-kb cytomegalovirus enhancer/chicken b-actin promoter, the woodchuck post-transcriptional regulatory element and the bovine growth hormone polyA. For immunohistochemistry, rAAV-Cre (1:1,000 diluted in 1 × PBS) was co-injected with rAAV-DIO-mGFP^47^ (1:1). This strategy was used to obtain single GCs labelled with mGFP. The rAAV-DIO-mGFP construct contains the mGFP sequence in an inverted orientation (3′ to 5′ direction) with loxP and lox2272 sites flanking it on each side, intercalated in opposing orientations (loxP>lox2272>^3′^mGFP^5′^<lox*P*<lox2272). Cre-recombinase expression then yields an inversion of the mGFP sequence and expression of mGFP. For voltage-clamp experiments of mIPSC recordings of GCs, rAAV-Cre was co-injected with rAAV-stopflox-Clomeleon^48^ in GABRB3^flox/flox^ mice to identify Cre-infected cells.

### Immunohistochemistry

To specifically analyse the expression and distribution of the β3-subunit, 3D immunohistochemistry was performed. In short, 21-day-old wild-type mice were stereotaxically injected with rAAV-Cre diluted 1:1,000 in 1 × PBS together with rAAV-DIO-mGFP mixed in a 1:1 ratio (for sparse expression) to identify single GCs. After 3–4 week expression, the animals were transcardially perfused using 4% paraformaldehyde (PFA). The brains were removed and free-floating 50-μm-thick horizontal slices of the OB were made using a vibratome. Antigen retrieval was made by incubating OB slices in 25-mM tri-sodium citrate (pH=7.4) in a warm water bath at 60 °C during 30 min. This temperature did not change fluorescence properties of GFP. Afterwards, the slices were incubated for 45 min at room temperature in vehicle buffer (5% normal goat serum, 0.5% bovine serum albumin fraction V and 0.3% Triton X-100, in 1 × PBS, pH=7.4) with 0.1% cold fish gelatin (blocking solution). The antibody against the β3-subunit (Novus Biologicals, cat. #: NB300-199) was carried in vehicle (1:1,000 dilution) and incubated overnight at 4 °C. After the third wash in vehicle (10 min each wash step), the alexa dye-conjugated secondary antibody (1:1,000 in vehicle; Life Technologies cat. #: A-21244) was incubated for 90 min at room temperature. The slices were washed three times in 1 × PBS, pH=7.4, and mounted on coverslips using self-made moviol. All incubation steps were performed under gentle incubation. For the triple labelling, the antibodies against VIAAT (SYSY, cat. # 131004) and gephyrin (kind gift from Professor Jochen Kuhse) were used.

### Quantification of β3-expression in the OB

Single-frame confocal images (1,476.2 μm × 1,476.2 μm with a pixel size of 1.44 μm) were used without additional processing. From each layer, squares with 100 × 100 pixels were selected and the average grey value in the GCL, EPL and GL was quantified (*n*=4, 4 regions per experiment). For each experiment the average grey value of the GCL was used to normalize the pixel intensity of the EPL and GL.

### Imaging

Images were acquired in a confocal microscope (Leica SP5, Leica, Germany) using a × 63 glycerol immersion objective (numeric aperture=1.3). For high-resolution image acquisition, 150-nm z steps were performed, with 1,024 × 1,024 or 2,048 × 2,048 pixels in the *xy* plane, with a field of view of 234.32 μm × 234.32 μm.

3D reconstructions were performed using the mGFP signal to do anatomical reconstructions of entire cells, cell bodies and dendrites. To analyse the cellular localization of the β3-subunit, VIAAT and gephyrin, the antibody signal was excised by image multiplication with the mGFP channel using the 3D immunohistochemistry approach^31^.

### Quantitative western blot analysis

Western blots were made from whole-OB protein extracts isolated in ice-cold artificial cerebrospinal fluid (see electrophysiology section). In total, OBs from six mice were used (three control and three rAAV-Cre injected) and two samples per mouse were analysed. The nuclear fraction was discarded by centrifugation at 800*g*, 20 min at 4 °C. The supernatants were loaded in self-made 5–16% gradient gels for electrophoresis^49^. Nitrocelulose membranes were used to blot the proteins. β3-subunit (Novus Biologicals, cat. #: NB300-199) was detected by using a goat anti-rabbit horseradish peroxidase-conjugated antibody (Sigma-Aldrich, cat. # P7899). For actin detection, a primary antibody horseradish peroxidase conjugated was used (Sigma-Aldrich, cat. # A3854). The peroxidase reaction was made using enhanced chemoluminescence (Thermo Scientific Pierce ECL Plus substrate, cat. # 32132). X-ray films were scanned in a conventional scanner (Canon). The grey images were further processed in ImageJ. Square selections of regions of interest were made, keeping the area constant across all measurements. Pixel intensities were used to calculate the ratios of actin and β3-subunit between wild-type and GABRB3^ΔGCL^ subjects.

### Quantification of Cre expression efficiency

To access the total number of nuclei (DAPI, Sigma-Aldrich cat. # D9542-1MG), total number of neural cells (NeuN, Millipore, cat. # MAB377) and total number of Cre-positive cells (Cre, Novagen, cat. # 69050), 4% PFA-fixed 50-μm-thick horizontal OB slices were used, that were obtained from mice sterotaxically injected with rAAV-Cre used in behaviour (*n*=3). Stacks of images (200 × 200 μm in the *xy* plane and 0.15 μm in *z* plane) of randomly selected regions in the GCL were acquired in a confocal microscope (Leica SP5). From each mouse, three to five slices were used and in each slice eight to ten regions were imaged. To count nuclei, maximal intensity projections of stacks of 10 images were used, using the ImageJ cell counter built-in function.

### Electrophysiology

OB horizontal slices (300-μm thick) were prepared from 6–10-week-old mice using a vibratome (Leica VT1200) while submerged in ice-cold oxygenated Ringer slicing solution (in mM): 125 NaCl, 2.5 KCl, 25 NaHCO_3_, 1.25 NaH_2_PO_4_, 3 myo-Inositol, 2 Na-pyruvate, 0.4 ascorbic acid, 0.1 CaCl_2_ and 3 MgCl_2_. The slices were transferred and incubated for ∼30 min in a 37 °C warm oxygenated bath solution (in mM): 125 NaCl, 2.5 KCl, 25 NaHCO_3_, 1.25 NaH_2_PO_4_, 2 CaCl_2_ and 1 MgCl_2_. All recordings were performed at 33–35 °C in bath solution.

Whole-cell recordings were established using an EPC9 amplifier (HEKA, Lambrecht, Germany). All recordings were made at 34±1 °C. To evaluate mIPSCs in GCs, voltage-clamp recordings were performed using pipettes with resistances ranging 4–5 MΩ, filled with (in mM) the following: 120 CsCl, 4 NaCl, 10 Na_2_-phosphocreatine, 10 HEPES, 0.1 EGTA, 10 Na-gluconate, 4 Mg-ATP, 0.3 Na_3_-GTP and 2.5 QX-314, pH=7.2 with CsOH. To the bath solution, 10 mM 6-cyano-7-nitroquinoxaline-2,3-dione (CNQX), 50 mM 2-amino-5-phosphonopentanoic acid (APV) and 1 μm tetrodotoxin (TTX) were added. Muscimol (100 μM in bath solution) was locally applied by using a second pipette (2–3 MΩ) positioned next to the GC soma, connected to a Picospritzer III (Parker Hannifin Corp., USA) to precisely control muscimol delivery (50 ms application). Current-clamp recordings were established from MCs to access recurrent inhibition. To induce a single AP, a 3-ms current pulse was used. To induce 20 consecutive APs, 3-ms current pulses interleaved with 8 ms of no-current injection were used. Pipettes had resistances of 3–4 MΩ, filled with (in mM): 135 K-gluconate, 10 HEPES, 10 Na_2_-phosphocreatine, 4 MgATP, 4 KCl and 0.3 Na_3_GTP, pH=7.2 adjusted with KOH. To access cell morphology, the dye alexa 594 hydrazide (100 μM) was added routinely in the intracellular solutions in all experiments.

The amount of current injected in MCs to fire a single AP was defined as threshold current. Cells that did not fire 20 consecutive APs at the maximal current injection possible using our amplifier were excluded from statistical analysis.

### Behavioural assessment

Behaviour experiments were conducted using a go/no-go operant conditioning scheme as previously published^19^,^34^. Male mice were kept separated in macrolon cages type II in a reverse light cycle (12–12 h), in a temperature- and humidity-controlled environment. Behaviour experiments were conducted in a dark room, corresponding to the night period. Behavioural pre-training, during which mice explored the set-up and learned that water can be obtained by licking at a metal tube placed in the odour port, started 3–4 weeks after the surgery and 2–3 days before the animals were kept under water restriction (by periods no longer than 12 h). The weight of the animals was strictly monitored and kept >85% of the initial body weight. The pre-training took 3–5 days and the behavioural training took 4–8 weeks. All animals tested were able to successfully learn the behaviour paradigm. Eight-channel semi-automated olfactometers^50^ (Knosys, Washington) and data acquisition were controlled through custom-programmed software, written in Igor Pro 6 (Wavemetrics Inc.). All odours were diluted to 1% in mineral oil and further diluted 1:20 in air in the olfactometers. Test odours (AA, EB, cineol, eugenol and binary mixtures thereof) were used to determine the reaction times. Odours were presented in a randomized manner, however, disabling more than five consecutive trials of the same stimulus. Bias towards any of the odours presented was avoided by counterbalancing two groups of mice.

The ODT was determined as described previously^34^ and is illustrated in Fig. 7a,b. A mouse self-initiates a trial by poking its head into the sampling port and breaking an infrared light beam. Mice need to wait 0.5 s (brown bar) at the sampling port for the odour onset to ensure that the odourant is present at the final valve at the appropriate concentration. Then, the odour is applied for 2 s. Mice stereotypically retracted their heads from the sampling port when unrewarded stimuli were presented (S−); however, they kept their heads inside the sampling port when rewarded stimuli were presented (S+) and immediately started licking at the reward tube to receive the water reward at the end of the trial (2.5 s total trial duration). Mice needed to lick during at least three 500-ms time bins (out of four in the 2-s trial time) to score a correct response. The sampling pattern (time when the head remained inside the sampling port) of S− stimuli was used to determine the ODT. Alternatively, the onset of licking for S+ trials could be used to determine ODT. After reaching >90% discrimination accuracy, an average curve was calculated from each 300S− and S+ trials, and bootstrapping statisitics^19^,^34^ was used to determine the first time point at which both curves differed significantly (*P*=0.05; first detect reaction time). This time was defined as the ODT. Hence, the ODT refers to a state after which mice have learned to discriminate an odour pair with high accuracy. In contrast, discrimination learning refers to the time when mice require to learn the discrimination of an odour pair from a naive state to highly accurate discrimination (>90% correct responses).

In the first experiment, GABRB3^lox/lox^ mice were used only by testing sham-injected (rAAV-eGFP) versus rAAV-Cre-injected mice. The second experiment was designed with WT animals (no floxed alleles) versus GABRB3^lox/lox^ animals. First, all animals went through the established behaviour protocol to see whether the presence of the loxP sites *per se* would change reaction times. In this experiment, before the test odours, cineol and eugenol were used for task habituation until the animals reached >80% odour discrimination accuracy. After this first behaviour round, all animals were injected with rAAV-Cre (same production batch) and reaction times were again accessed.

### Data analysis

Immunohistochemistry data were analysed in ImageJ. The procedure is illustrated in Supplementary Figs 2 and 3. First, the data were filtered using a two-dimensonal (2D) anisotropic diffusion filter. To reduce noise, a mask at the centre (180°) of the Fourier frequency domain of the images was applied by setting the pixel intensity to zero in each image, within a radius of 5 pixels. Afterwards, a Fourier-based bandpass filter was used to smoothen the signal. Colocalization analysis was performed with the imageJ colocalization finder plugin, and 3D visualizations were generated using the ImageJ 3D viewer plugin. Snapshots of the 3D visualizations are presented in the figures.

Electrophysiology and behaviour data analysis were performed using self-made custom software written in Igor Pro6 (Wavemetrics Inc).

Data are reported as mean±s.e.m. *t*-test refers to the unpaired two-sided Student's *t*-test unless otherwise noted. For each experiment, the number of samples tested was sufficiently big to match the statistical requirements for population representation and robust statistical analysis. Each experimental procedure was repeated at least three times to evaluate the coherency of results across experiments, and data were pulled together in the statistical analysis.

## Additional information

**How to cite this article:** Nunes, D. & Kuner, T. Disinhibition of olfactory bulb granule cells accelerates odour discrimination in mice. *Nat. Commun.* 6:8950 doi: 10.1038/ncomms9950 (2015).

## Supplementary Material

Supplementary Information Supplementary Figures 1-5 and Supplementary Reference

## References

[b1] Didier A. . A dendrodendritic reciprocal synapse provides a recurrent excitatory connection in the olfactory bulb. Proc. Natl Acad. Sci. USA 98, 6441–6446 (2001). 11353824 10.1073/pnas.101126398 PMC33487

[b2] Nicoll R. A. Inhibitory mechanisms in the rabbit olfactory bulb: dendrodendritic mechanisms. Brain Res. 14, 157–172 (1969). 5783107 10.1016/0006-8993(69)90037-7

[b3] Aungst J. L. . Centre-surround inhibition among olfactory bulb glomeruli. Nature 426, 623–629 (2003). 14668854 10.1038/nature02185

[b4] Shepherd G. M. Perspectives on olfactory processing, conscious perception, and orbitofrontal cortex. Ann. N Y Acad. Sci. 1121, 87–101 (2007). 17872397 10.1196/annals.1401.032

[b5] Arevian A. C., Kapoor V. & Urban N. N. Activity-dependent gating of lateral inhibition in the mouse olfactory bulb. Nat. Neurosci. 11, 80–87 (2008). 18084286 10.1038/nn2030 PMC2720685

[b6] Friedrich R. W. & Laurent G. Dynamic optimization of odor representations by slow temporal patterning of mitral cell activity. Science 291, 889–894 (2001). 11157170 10.1126/science.291.5505.889

[b7] Nusser Z., Kay L. M., Laurent G., Homanics G. E. & Mody I. Disruption of GABA(A) receptors on GABAergic interneurons leads to increased oscillatory power in the olfactory bulb network. J. Neurophysiol. 86, 2823–2833 (2001). 11731539 10.1152/jn.2001.86.6.2823

[b8] Schild D. Principles of odor coding and a neural network for odor discrimination. Biophys. J. 54, 1001–1011 (1988). 3233263 10.1016/S0006-3495(88)83038-8 PMC1330413

[b9] Mori K., Nagao H. & Yoshihara Y. The olfactory bulb: coding and processing of odor molecule information. Science 286, 711–715 (1999). 10531048 10.1126/science.286.5440.711

[b10] Leon M. & Johnson B. A. Olfactory coding in the mammalian olfactory bulb. Brain Res. Brain Res. Rev. 42, 23–32 (2003). 12668289 10.1016/s0165-0173(03)00142-5

[b11] Egger V., Svoboda K. & Mainen Z. F. Mechanisms of lateral inhibition in the olfactory bulb: efficiency and modulation of spike-evoked calcium influx into granule cells. J. Neurosci. 23, 7551–7558 (2003). 12930793 10.1523/JNEUROSCI.23-20-07551.2003 PMC6740749

[b12] Margrie T. W. & Schaefer A. T. Theta oscillation coupled spike latencies yield computational vigour in a mammalian sensory system. J. Physiol. 546, 363–374 (2003). 12527724 10.1113/jphysiol.2002.031245 PMC2342519

[b13] Phillips C. G., Powell T. P. & Shepherd G. M. Responses of mitral cells to stimulation of the lateral olfactory tract in the rabbit. J. Physiol. 168, 65–88 (1963). 14056493 10.1113/jphysiol.1963.sp007178 PMC1359410

[b14] Rall W., Shepherd G. M., Reese T. S. & Brightman M. W. Dendrodendritic synaptic pathway for inhibition in the olfactory bulb. Exp. Neurol. 14, 44–56 (1966). 5900523 10.1016/0014-4886(66)90023-9

[b15] Price J. L. & Powell T. P. The synaptology of the granule cells of the olfactory bulb. J. Cell Sci. 7, 125–155 (1970). 5476853 10.1242/jcs.7.1.125

[b16] Wellis D. P. & Kauer J. S. GABAA and glutamate receptor involvement in dendrodendritic synaptic interactions from salamander olfactory bulb. J. Physiol. 469, 315–339 (1993). 7903696 10.1113/jphysiol.1993.sp019816 PMC1143873

[b17] Yokoi M., Mori K. & Nakanishi S. Refinement of odor molecule tuning by dendrodendritic synaptic inhibition in the olfactory bulb. Proc. Natl Acad. Sci. USA 92, 3371–3375 (1995). 7724568 10.1073/pnas.92.8.3371 PMC42168

[b18] Isaacson J. S. & Strowbridge B. W. Olfactory reciprocal synapses: dendritic signaling in the CNS. Neuron 20, 749–761 (1998). 9581766 10.1016/s0896-6273(00)81013-2

[b19] Abraham N. M. . Synaptic inhibition in the olfactory bulb accelerates odor discrimination in mice. Neuron 65, 399–411 (2010). 20159452 10.1016/j.neuron.2010.01.009 PMC6366558

[b20] Levine C. & Marcillo A. Regarding several points of doubt of the structure of the olfactory bulb: as described by T. Blanes. Anat. Rec. (Hoboken) 291, 751–762 (2008). 18384121 10.1002/ar.20680

[b21] Schneider S. P. & Macrides F. Laminar distributions of internuerons in the main olfactory bulb of the adult hamster. Brain Res. Bull. 3, 73–82 (1978). 630423 10.1016/0361-9230(78)90063-1

[b22] Pressler R. T. & Strowbridge B. W. Blanes cells mediate persistent feedforward inhibition onto granule cells in the olfactory bulb. Neuron 49, 889–904 (2006). 16543136 10.1016/j.neuron.2006.02.019

[b23] Schoppa N. E. A novel local circuit in the olfactory bulb involving an old short-axon cell. Neuron 49, 783–784 (2006). 16543124 10.1016/j.neuron.2006.03.005

[b24] Laurie D. J., Wisden W. & Seeburg P. H. The distribution of thirteen GABAA receptor subunit mRNAs in the rat brain. III. Embryonic and postnatal development. J. Neurosci. 12, 4151–4172 (1992). 1331359 10.1523/JNEUROSCI.12-11-04151.1992 PMC6576006

[b25] Wisden W., Laurie D. J., Monyer H. & Seeburg P. H. The distribution of 13 GABAA receptor subunit mRNAs in the rat brain. I. Telencephalon, diencephalon, mesencephalon. J. Neurosci. 12, 1040–1062 (1992). 1312131 10.1523/JNEUROSCI.12-03-01040.1992 PMC6576059

[b26] Pirker S., Schwarzer C., Wieselthaler A., Sieghart W. & Sperk G. GABA(A) receptors: immunocytochemical distribution of 13 subunits in the adult rat brain. Neuroscience 101, 815–850 (2000). 11113332 10.1016/s0306-4522(00)00442-5

[b27] Huntsman M. M., Munoz A. & Jones E. G. Temporal modulation of GABA(A) receptor subunit gene expression in developing monkey cerebral cortex. Neuroscience 91, 1223–1245 (1999). 10391431 10.1016/s0306-4522(98)00713-1

[b28] Hentschke H. . Altered GABAA,slow inhibition and network oscillations in mice lacking the GABAA receptor beta3 subunit. J. Neurophysiol. 102, 3643–3655 (2009). 19846622 10.1152/jn.00651.2009 PMC2804419

[b29] Ferguson C. . New insight into the role of the beta3 subunit of the GABAA-R in development, behavior, body weight regulation, and anesthesia revealed by conditional gene knockout. BMC Neurosci. 8, 85 (2007). 17927825 10.1186/1471-2202-8-85 PMC2100059

[b30] Miralles C. P., Li M., Mehta A. K., Khan Z. U. & De Blas A. L. Immunocytochemical localization of the beta(3) subunit of the gamma-aminobutyric acid(A) receptor in the rat brain. J. Comp. Neurol. 413, 535–548 (1999). 10495441

[b31] Dondzillo A. . Targeted three-dimensional immunohistochemistry reveals localization of presynaptic proteins Bassoon and Piccolo in the rat calyx of Held before and after the onset of hearing. J. Comp. Neurol. 518, 1008–1029 (2010). 20127803 10.1002/cne.22260

[b32] Eyre M. D., Kerti K. & Nusser Z. Molecular diversity of deep short-axon cells of the rat main olfactory bulb. Eur. J. Neurosci. 29, 1397–1407 (2009). 19344330 10.1111/j.1460-9568.2009.06703.x

[b33] Belelli D., Peden D. R., Rosahl T. W., Wafford K. A. & Lambert J. J. Extrasynaptic GABAA receptors of thalamocortical neurons: a molecular target for hypnotics. J. Neurosci. 25, 11513–11520 (2005). 16354909 10.1523/JNEUROSCI.2679-05.2005 PMC6726038

[b34] Abraham N. M. . Maintaining accuracy at the expense of speed: stimulus similarity defines odor discrimination time in mice. Neuron 44, 865–876 (2004). 15572116 10.1016/j.neuron.2004.11.017

[b35] Shimshek D. R. . Enhanced odor discrimination and impaired olfactory memory by spatially controlled switch of AMPA receptors. PLoS Biol. 3, e354 (2005). 16216087 10.1371/journal.pbio.0030354 PMC1255741

[b36] Labarrera C., London M. & Angelo K. Tonic inhibition sets the state of excitability in olfactory bulb granule cells. J. Physiol. 591, 1841–1850 (2013). 23318869 10.1113/jphysiol.2012.241851 PMC3624854

[b37] Korpi E. R., Mattila M. J., Wisden W. & Luddens H. GABA(A)-receptor subtypes: clinical efficacy and selectivity of benzodiazepine site ligands. Ann. Med. 29, 275–282 (1997). 9375983 10.3109/07853899708999348

[b38] Wafford K. A. . Functional characterization of human gamma-aminobutyric acidA receptors containing the alpha 4 subunit. Mol. Pharmacol. 50, 670–678 (1996). 8794909

[b39] Olsen R. W. & Sieghart W. International Union of Pharmacology. LXX. Subtypes of gamma-aminobutyric acid(A) receptors: classification on the basis of subunit composition, pharmacology, and function. Update. Pharmacol. Rev. 60, 243–260 (2008). 18790874 10.1124/pr.108.00505 PMC2847512

[b40] Olsen R. W. & Sieghart W. GABA A receptors: subtypes provide diversity of function and pharmacology. Neuropharmacology 56, 141–148 (2009). 18760291 10.1016/j.neuropharm.2008.07.045 PMC3525320

[b41] Viitanen T., Ruusuvuori E., Kaila K. & Voipio J. The K+-Cl cotransporter KCC2 promotes GABAergic excitation in the mature rat hippocampus. J. Physiol. 588, 1527–1540 (2010). 20211979 10.1113/jphysiol.2009.181826 PMC2876807

[b42] Nagayama S., Homma R. & Imamura F. Neuronal organization of olfactory bulb circuits. Front. Neural Circ. 8, 98 (2014). 10.3389/fncir.2014.00098 PMC4153298 25232305

[b43] Zaborszky L., Carlsen J., Brashear H. R. & Heimer L. Cholinergic and GABAergic afferents to the olfactory bulb in the rat with special emphasis on the projection neurons in the nucleus of the horizontal limb of the diagonal band. J. Comp. Neurol. 243, 488–509 (1986). 3512629 10.1002/cne.902430405

[b44] Kato H. K., Gillet S. N., Peters A. J., Isaacson J. S. & Komiyama T. Parvalbumin-expressing interneurons linearly control olfactory bulb output. Neuron 80, 1218–1231 (2013). 24239124 10.1016/j.neuron.2013.08.036 PMC3884945

[b45] Kato H. K., Chu M. W., Isaacson J. S. & Komiyama T. Dynamic sensory representations in the olfactory bulb: modulation by wakefulness and experience. Neuron 76, 962–975 (2012). 23217744 10.1016/j.neuron.2012.09.037 PMC3523713

[b46] Wellis D. P. & Kauer J. S. GABAergic and glutamatergic synaptic input to identified granule cells in salamander olfactory bulb. J. Physiol. 475, 419–430 (1994). 8006826 10.1113/jphysiol.1994.sp020082 PMC1160394

[b47] Sohal V. S., Zhang F., Yizhar O. & Deisseroth K. Parvalbumin neurons and gamma rhythms enhance cortical circuit performance. Nature 459, 698–702 (2009). 19396159 10.1038/nature07991 PMC3969859

[b48] Berglund K. . Imaging synaptic inhibition throughout the brain via genetically targeted Clomeleon. Brain Cell. Biol. 36, 101–118 (2008). 18850274 10.1007/s11068-008-9031-x PMC2674236

[b49] Bolt M. W. & Mahoney P. A. High-efficiency blotting of proteins of diverse sizes following sodium dodecyl sulfate-polyacrylamide gel electrophoresis. Anal. Biochem. 247, 185–192 (1997). 9177676 10.1006/abio.1997.2061

[b50] Bodyak N. & Slotnick B. Paerformance of mice in an automated olfactometer: odor detection, discrimination and odor memory. Chem. Senses 24, 637–645 (1999). 10587496 10.1093/chemse/24.6.637

