# Supplementary material for: Disinhibition of olfactory bulb granule cells accelerates odour discrimination in mice
Source: Nat Commun. 2015 Nov 23;6:8950. doi: 10.1038/ncomms9950 (PMC4673882; doi:10.1038/ncomms9950)
Supplement: Supplementary Information — Supplementary Figures 1-5 and Supplementary Reference [file ncomms9950-s1.pdf]

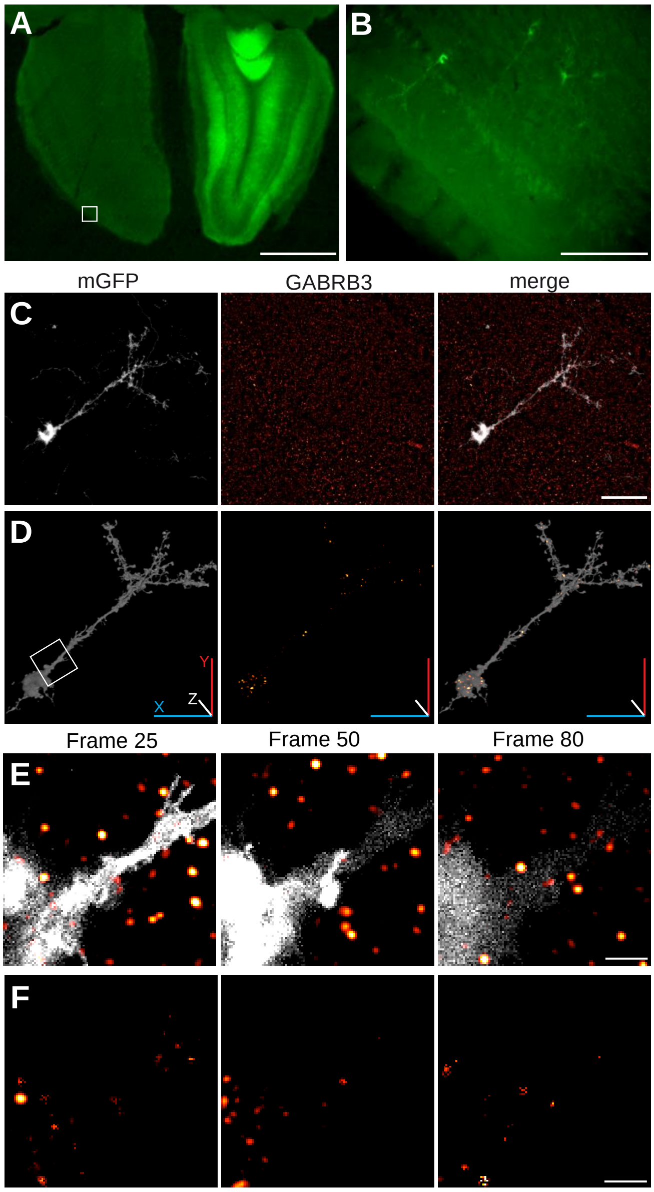

Supplementary Figure 1: Sparse labelling and 3D-immunohistochemistry[1]

Anatomical reconstructions of entire GCs were obtained by sparsely labeling GCs with mGFP and immunostaining against the β3-subunit. The mGFP signal was subsequently used to excise the β3-subunit immuno-signals residing within the labeled GC.

(A) Comparison of OBs infected with rAAV-Cre (1:1000) and rAAV-DIO-mGFP (left) or rAAV-mGFP (right). Individual GCs can not be identified at low magnification (mostly background fluorescence visible, left) while dense

expression is evident (right). Wide-field epi-fluorescence microscopy. Scale bar: 1mm.

(B) Magnification of the region delimited in (A). Individual GCs are detectable at higher magnification. Wide-field epi-fluorescence microscopy. Scale bar: 200 µm.

(C) Individual GC and corresponding β3-immunosignal shown as maximal intensity projections calculated from 101 confocal imageframes obtained at maximal confocal resolution using sequential scanning. The panel on the right shows an overlay of both datasets. The GC is embedded in a dense punctate labeling pattern reflecting postsynaptic densities of GABAergic synapses in the GCL and ECL. Scale bar: 50 µm.

(D) 3D reconstruction of the cell shown in (C) using the ImageJ 3D-viewer plugin. GABRB3 immuno-signals residing within the GC were excised by frame-wise multiplication of the two image stacks (pixels within the GC are retained, all others are set to 0). The Cartesian axes X (blue), Y (red) and Z (white) are indicated. They do not represent scale bars (image width approximately 200 µm).

(E) Single frames of the region marked in D demonstrate that β3-subunit clusters are well resolved and mostly separated from each other (the low magnification images of ß3-immunoreactivity shown in C and Fig. 1A may suggest that such dense labeling will not allow separation and segregation of individual clusters, however, this is due to the limited resolution of the overview images). For processing of confocal images see Supplementary Figs. 2,3. Some clusters overlap with the GC volume. Scale bars: 5 µm.

(F) Frame-by-frame channel multiplication ensures that only the β3-subunit clusters residing inside the labeled GC are isolated (Panels correspond to E). Some of the small clusters shown in F arise from large clusters that are in close vicinity to the GC and therefore some overlapping pixels are retained. However, these clusters can not be considered part of the GC because the major part of their volume resides outside the GC volume. Scale bars: 5 µm.

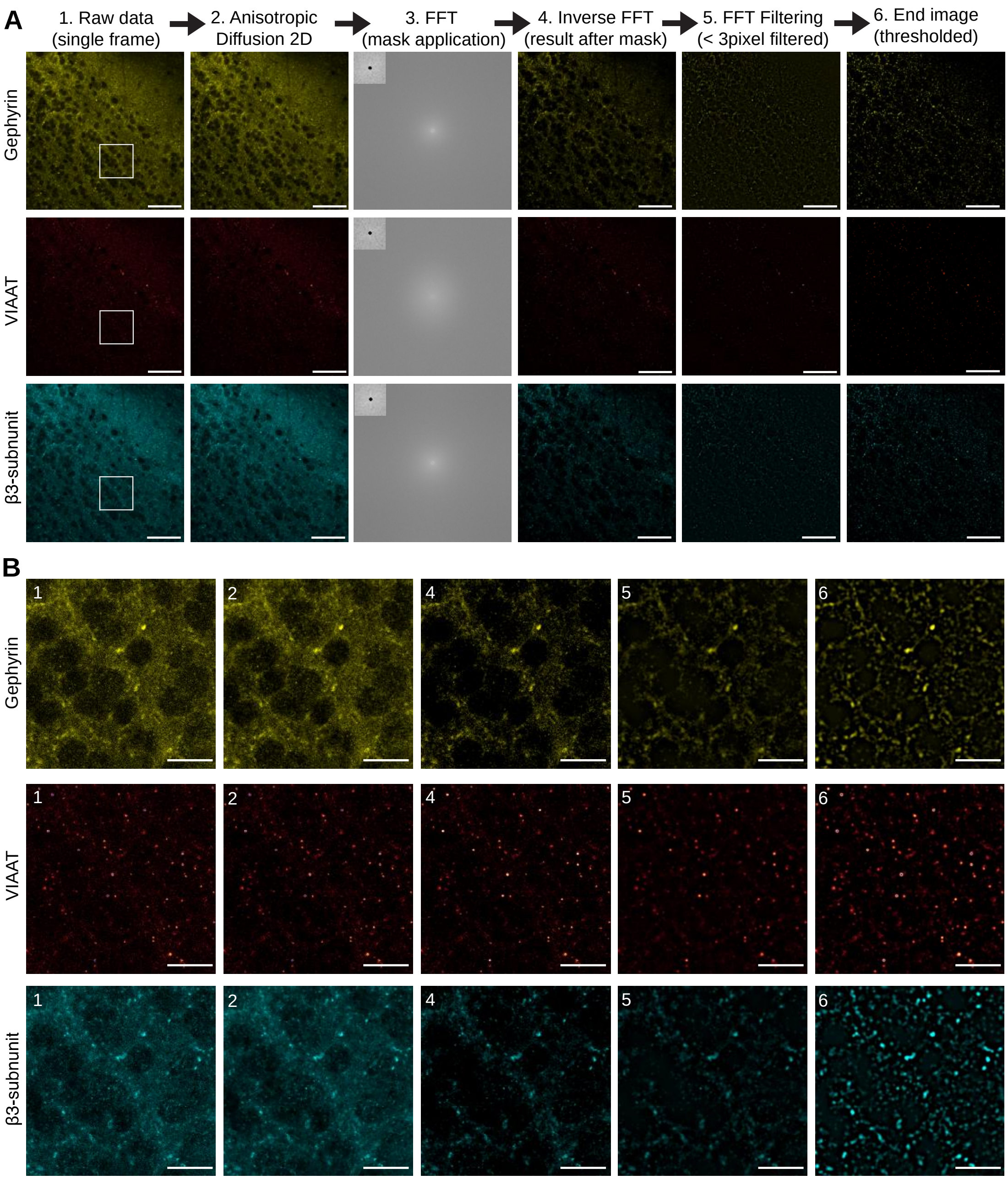

Supplementary Figure 2. Processing of immunostainings.

(A) Each channel was filtered frame by frame. The raw data was acquired at high confocal resolution (XY: 234.32 µm x 234.32 µm, pixel size = 0.114 µm) using a 63x glycerol objective (NA=1.3). Z-steps were 0.15 µm per frame. Acquired raw data (1) was filtered using an anisotropic diffusion 2D filter (2). Afterwards, a 5 pixels mask was applied (3) in the centre (small inset in 3) of the image power spectrum obtained using the Forward Fourier Transform (FFT) algorithm. Upon application of the inverse of the FFT (4), most noise was removed from the image. Nevertheless, we assumed that structures

smaller than 3 continuous pixels were under the confocal resolution limit, so a FFT based bandpass filter was applied to remove these structures (5). The final images (6) were obtained by thresholding the immunosignal. Scale bars: 50 µm.

(B) Magnification of regions highlighted in A to show image filtering on the level of individual clusters. Scale bars: 10µm.

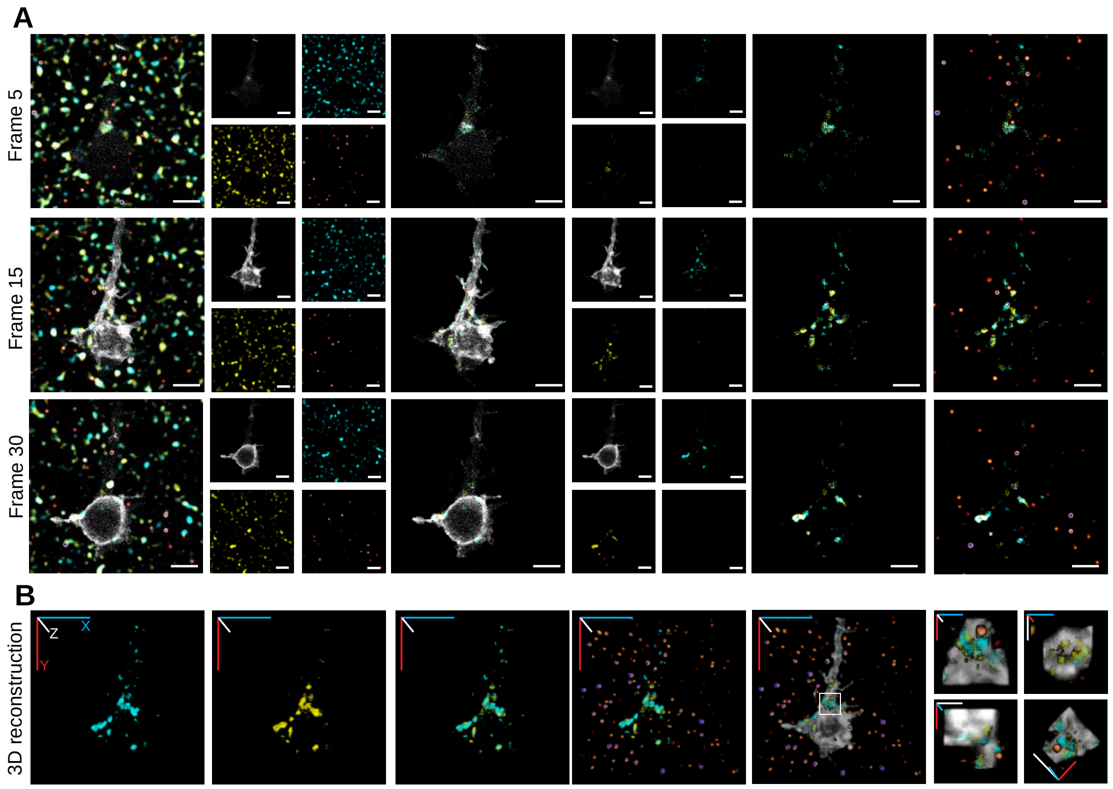

Supplementary Figure 3. Conversion of confocal stacks into 3D reconstructions.

(A) Three example frames of a confocal stack containing the mGFP signal delineating the GC (grey) as well as β3 (cyan), gephyrin (yellow) and VIAAT (glow over palette) immunosignals. The left set of panels shows superpositions of all four channels and the respective individual image frames. The central set of panels shows the immunosignals residing within the GC, obtained through frame-by-frame multiplication. The right set of panels shows superpositions of ß3 and gephyrin immunosignals from within the GC and the same with surrounding VIAAT immunoreactivities. The Pearson's coefficient of co-localization for β3-subunit and gephyrin was 0.91 in frame 5, 0.87 in frame 15 and 0.92 in frame 30. Over 70 image frames obtained for this cell, it was 0.88, supporting the strong co-localization shown in Fig. 2. Scale bars: 5 µm

(B) 3D-reconstruction (ImageJ plugin 3D-viewer) of 70 image frames partially shown in A. The β3-subunit (cyan) highly colocalizes with gephyrin, but not VIAAT. The small panels on the right illustrate the region of interest shown in the preceding panel at different angles. Cartesian axes X (blue), Y (red) and Z

(white) do not represent scale bars due to non-isotropic pixel size. The dimensions similar to A.

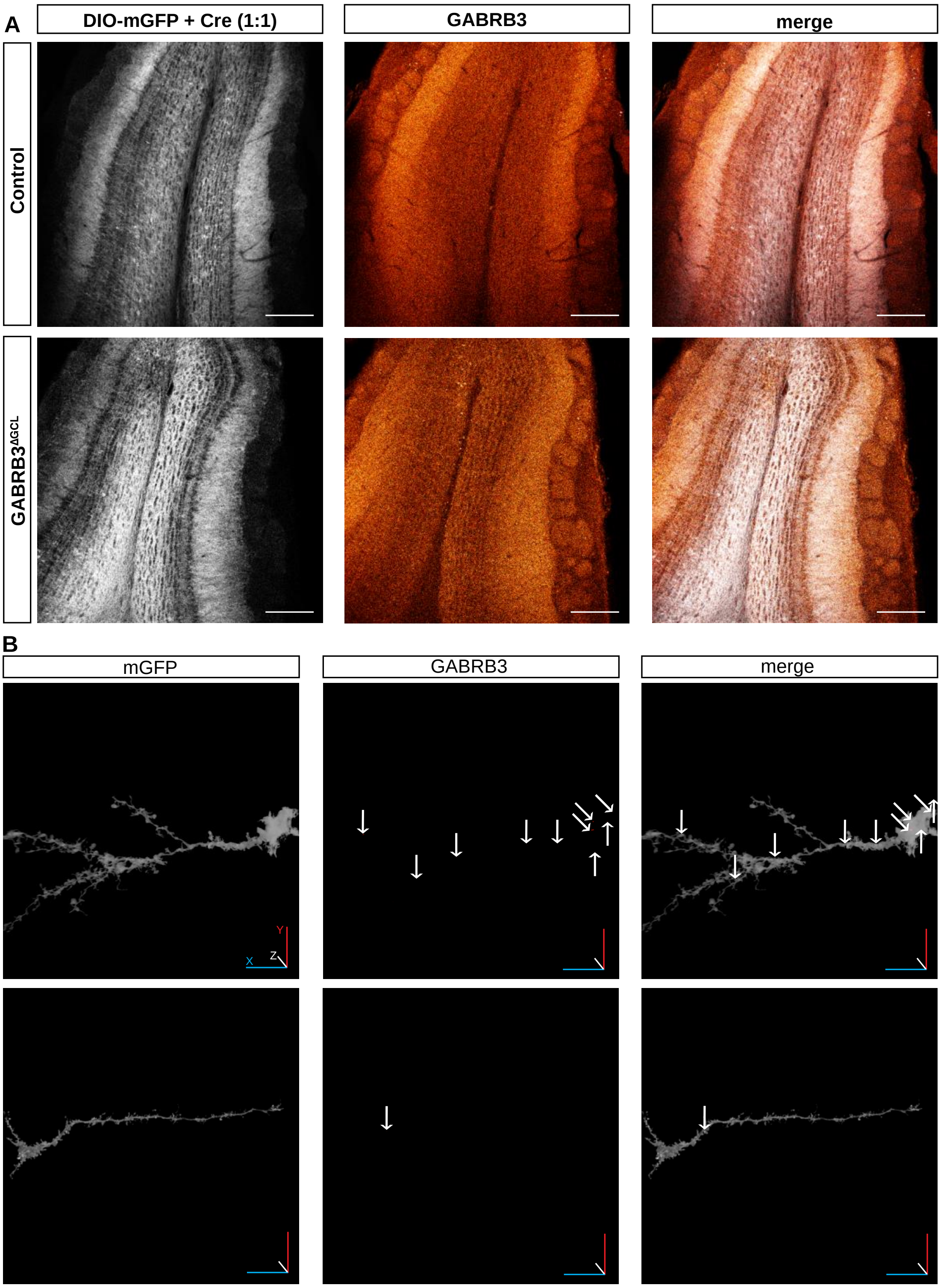

Supplementary Figure 4. Reduction of the β3-subunit immunosignal intensity in the GCL.

(A) Low magnification single confocal planes of control mice and GABRB3$^{lox/lox}$ mice co-injected with rAAV-DIO-mGFP and rAAV-Cre (1:1) in the OB. mGFP signal showing GCs (left panels), β3-subunit (central panels)

and merge of both channels (right panels) 3 weeks after Cre expression. Scale bars: 250 µm

(B) Further examples of GCs with deleted ß3-subunits (see legend to Fig. 4C). Arrows point to the small remaining signals that are within the GCs (glow over palette). Cartesian axes X (blue), Y (red) and Z (white). Entire GC images were acquired at high confocal resolution (pixel size=0.229 µm) including 84 (upper cell) an 104 image frames (lower cell). The width of the image is approximately 235 µm.

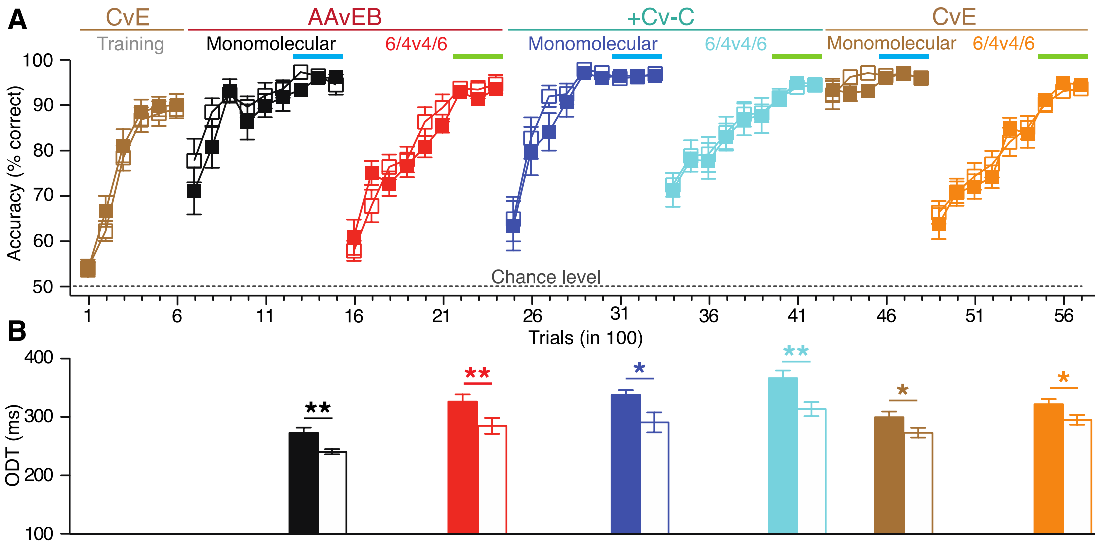

Supplementary Figure 5: Odor discriminations at different levels of difficulty are accelerated in GABRB3$^{\Delta GCL}$ mice

(A) Learning curves. Cineol and eugenol (CvE) were first used for task habituation. Test odors, amyl acetate and ethyl butyrate (AAvEB), (+)-Carvone and (-)-Carvone (+Cv-C), Cineol and Eugenol (CvE) and their binary mixtures in a 60:40 ratio (6/4v4/6), diluted to 1% in mineral oil were used to measure the discrimination times. Learning curves of control (n=12) and GABRB3$^{\Delta GCL}$ (n=12) mice did not differ in all odor pairs tested (mean±SEM; ANOVA; p>0.05). The colored bars (simple odors light blue and binary mixtures light green) depict the trials used for determining the odor discrimination times.

(B) Odor discrimination times for all odor pairs tested differed between control (n=12) and GABRB3$^{\Delta GCL}$ (n=12) mice. AAvEB and its binary mixture were the fastest discriminated odors (simple: control 274±9 ms, GABRB3$^{\Delta GCL}$ 241±4 ms, **p<0.01; binary mixture: control 330±12 ms, GABRB3$^{\Delta GCL}$ 290±14 ms, *p<0.05; mean±SEM), while +Cv-C took the longest to be discriminated (simple: control 338±8.3 ms, GABRB3$^{\Delta GCL}$ 291±17 ms, *p<0.05; binary mixture: control 367±13 ms, GABRB3$^{\Delta GCL}$ 313±12 ms, **p<0.01; mean±SEM), respectively. CvE and its binary mixture had intermediate discrimination times (simple: control 300±9.7 ms, GABRB3$^{\Delta GCL}$ 270±8 ms, *p=0.05; binary mixture: control 320±9 ms, GABRB3$^{\Delta GCL}$ 300±12 ms, *p<0.05; mean±SEM).

Supplementary References

1 Dondzillo, A. *et al.* Targeted three-dimensional immunohistochemistry reveals localization of presynaptic proteins Bassoon and Piccolo in the rat calyx of Held before and after the onset of hearing. *The Journal of comparative neurology* **518**, 1008-1029, doi:10.1002/cne.22260 (2010).
